# Review and Selection of Online Resources for Carers of Frail Adults or Older People in Five European Countries: Mixed-Methods Study

**DOI:** 10.2196/14618

**Published:** 2020-06-17

**Authors:** Roberta Papa, Areti Efthymiou, Giovanni Lamura, Flavia Piccinini, Giulia Onorati, Evridiki Papastavrou, Theologia Tsitsi, Giulia Casu, Licia Boccaletti, Alessandra Manattini, Rita Seneca, Carlos Vaz de Carvalho, Rita Durão, Francesco Barbabella, Frida Andréasson, Lennart Magnusson, Elizabeth Hanson

**Affiliations:** 1 Centre for Socio-Economic Research on Ageing IRCCS INRCA Ancona Italy; 2 Department of Nursing Faculty of Health Sciences Cyprus University of Technology Limassol Cyprus; 3 Department of Psychology University of Bologna Bologna Italy; 4 Anziani e Non Solo – social cooperative Carpi Italy; 5 Virtual Campus, Lda Porto Portugal; 6 Department of Health and Caring Sciences Linnaeus University Kalmar Sweden; 7 Swedish Family Care Competence Centre Kalmar Sweden

**Keywords:** informal carers, mobile apps, websites, usability, reliability

## Abstract

**Background:**

Informal carers have a crucial role in the care of older people, but they are at risk of social isolation and psychological exhaustion. Web-based services like apps and websites are increasingly used to support informal carers in addressing some of their needs and tasks, such as health monitoring of their loved ones, information and communication, and stress management. Despite the growing number of available solutions, the lack of knowledge or skills of carers about the solutions often prevent their usage.

**Objective:**

This study aimed to review and select apps and websites offering functionalities useful for informal carers of frail adults or older people in 5 European countries (Cyprus, Greece, Italy, Portugal, and Sweden).

**Methods:**

A systematic online search was conducted from January 2017 to mid-March 2017 using selected keywords, followed by an assessment based on a set of commonly agreed criteria and standardized tools. Selected resources were rated and classified in terms of scope. Focus groups with informal carers were conducted to validate the list and the classification of resources. The activities were conducted in parallel in the participating countries using common protocols and guidelines, a standardization process, and scheduled group discussions.

**Results:**

From a total of 406 eligible resources retrieved, 138 apps and 86 websites met the inclusion criteria. Half of the selected resources (109/224, 48.7%) were disease-specific, and the remaining resources included information and utilities on a variety of themes. Only 38 resources (38/224, 17.0%) were devoted specifically to carers, addressing the management of health disturbances and diseases of the care recipient and focusing primarily on neurodegenerative diseases. Focus groups with the carers showed that almost all participants had no previous knowledge of any resource specifically targeting carers, even if interest was expressed towards carer-focused resources. The main barriers for using the resources were low digital skills of the carers and reliability of health-related apps and websites. Results of the focus groups led to a new taxonomy of the resources, comprising 4 categories: carer’s wellbeing, managing health and diseases of the care recipient, useful contacts, and technologies for eldercare.

**Conclusions:**

The review process allowed the identification of online resources of good quality. However, these resources are still scarce due to a lack of reliability and usability that prevent users from properly benefiting from most of the resources. The involvement of end users provided added value to the resource classification and highlighted the gap between the potential benefits from using information and communication technologies and the real use of online resources by carers.

## Introduction

Informal carers are people who provide unpaid care to someone with a chronic illness, disability, or other long-lasting health or care needs outside of a professional or formal framework [1]. They represent an inherent and indispensable component of current health and social care provision across Europe, providing 80% of all long-term care [2]. Caring can be highly rewarding, but also demanding, resulting in social isolation, physical exhaustion, and psychological exhaustion, including anxiety, depression, frustration, anger, guilt, grief, stigma, and difficulties in reconciliation of work and care responsibilities [3-5]. Providing care for over 10 years and more than 40 hours per week is not a rare phenomenon, and it affects not only the carers’ physical, emotional, social, spiritual, and financial wellbeing [6] but also the quality of the care provision itself [5]. A recent estimate of the outstanding role of informal care globally, based on the prevalence and incidence of noncommunicable diseases, has highlighted that for one care recipient, there are at least 3 carers [7]. Informal care is common throughout Europe, although with different characteristics [8].

Advances in clinical research and technological innovation in health care have opened new horizons in care provision and the support of vulnerable groups, and relevant solutions are offered both through care service providers and at the patient’s home [9]. Support services based on information and communication technologies (ICTs), such as interactive services, psychoeducational and stress management programs, carers’ platforms, e-learning courses, telemedicine, and telehealth [10-18], have the potential to support informal carers in their daily tasks [19]. Nowadays, terms such as electronic health (eHealth) and mobile health (mHealth) are gaining increased attention in the research community, making their appearance in published papers for over a decade now [20]. According to the Global Observatory for eHealth [21], mHealth is defined as “a medical and public health practice supported by mobile devices, such as mobile phones, patient monitoring devices, personal digital assistants, and other wireless devices.” Mobile devices can be of great advantage for carers as they are widely available and normally easier to use than PCs. They are also user-friendly and allow handy access to internet-based applications. It is worth mentioning that, in 2008, only 50% of the world population owned a smartphone [22], while in 2016, mobile web browsing overtook PCs for the first time [23].

Currently, there are apps already available on the market that can be useful for carers at any time, in any context, and for a variety of tasks. A taxonomy recently presented by Grossman et al [24] identified 8 app categories: information and resources (eg, disease information, videos, databases); useful resources for reminding of tasks and deadlines (eg, activity monitoring, personal organizer, medication reminders, diaries); support for carers (eg, support groups and chats); safety apps (eg, GPS, alarms, reminders); communication with family, friends, and professionals (eg, sharing task calendars, social networking, email, chat); care recipients’ activities (eg, reminiscence for persons with cognitive impairment, music, recreational activities, memory aids); personal health record tracking; and problem-solving solutions (eg, managing behavioral disorders).

Even though carers could benefit from many free or low-cost apps already offered on the market, some barriers prevent their use. Carers are often not aware of the apps, do not know how to install or use them on their mobile devices, or have not realized the potential benefits they could gain by using them [25-27]. Several factors are associated with the use of health-related web-based services and interventions among carers and might include the accessibility of the internet and related equipment, carers’ personal characteristics, social network and support, carers’ beliefs, duration of care, and type of web-based use (ie, reflective or passive use) [18,28]. The presence of a vast multitude of health-related apps and websites can be challenging, as it might be difficult to find reliable resources [29,30], especially for users with lower levels of eHealth literacy or mHealth literacy [31,32]. Digital skills training programs for carers are mainly available through projects, and it is not easy to find relevant publications apart from press releases and training curricula on project websites or carer associations.

Taking these considerations into account, the “Apps4Carers” study, funded by the Erasmus+ program in 2016-2018, aimed to overcome the barriers that currently limit informal carers and their care recipients from fully benefiting from learning, accessing information, and social participation opportunities offered via mobile devices. The specific objectives of this project were to: (1) select (among those already available on the market for free or at a very low cost) online resources (ie, apps and websites) offering functionalities useful for informal carers; (2) develop training content and methodologies to empower carers to use these resources; (3) provide informal carers with ICT skills for using mobile devices and online resources; (4) develop a mobile app to be used as a compact, usable, and informative library of the selected mobile resources dedicated to carers. In order to reach a greater impact and enhance transferability of the results, these aims were pursued transnationally in 5 countries (Cyprus, Greece, Italy, Portugal, Sweden), because caring is a European-wide issue, and the presence of different European country contexts, such as southern European and Scandinavian contexts, allowed the project outcomes to be tested in different cultural and socioeconomic contexts. This paper focused on the first stage of the project, namely the review and selection of the mobile resources to be included in the mobile app, through a mixed-methods approach based on a review and focus groups carried out in the involved countries.

## Methods

### Design

We used a mixed-methods approach with a sequential design. First, we searched, reviewed, and selected available online resources based on a set of criteria, followed by quantitative data collection. Then, we “validated” this list with a qualitative study (ie, focus groups) involving the end users, such as the carers. The quantitative study aimed to identify the available resources for carers and explore which features they have, which needs they address, and how usable and reliable they are. The qualitative study was used to check if the selected resources were known by the carers, if they might be useful for them, and if their classification was clear. These two phases were linked to the development of the Apps4Carers app [33], which has been designed to be an online, easy-to-use library including a peer-reviewed set of resources organized by and covering main carers’ needs. Indeed, the definition of the search strategy and the selection criteria aimed to obtain a list of resources as comprehensive as possible and of acceptable quality.

For the purpose of this study, we considered only carers aged ≥18 years and both adults and older persons as care recipients.

### Search Strategy

We used a systematic approach to search online resources. In order to find apps and websites useful for carers’ tasks, 3 eligibility criteria were defined: target group, scope, and disease/condition. Regarding the target group, the search was aimed at finding resources specifically designed for carers. However, after a preliminary explorative search carried out in participating countries yielded few results, we decided to cover missing aspects considering eligible resources designed to facilitate the self-management of a disease by the patient and those not related to a specific disease or target group but supporting relevant tasks. The resources had to address the most important areas of needs and preferences of informal carers [34], such as care planning and management, information and microlearning, and communication and social inclusion, referred to as the scope. Finally, the search was focused on the major chronic diseases/conditions of the older population [35-37] associated with carer burden [38-44], selected through a rapid review of the literature. Thus, we included the following 11 chronic conditions: cardiovascular diseases, stroke, respiratory diseases, mental illness, neurodegenerative diseases, cancer, digestive diseases, sensory organ diseases, musculoskeletal diseases, diabetes, and urinary incontinence. The review and selection of resources were conducted in parallel in 5 countries (Italy, Greece, Cyprus, Portugal, Sweden) from January 2017 to mid-March 2017. We used the Google search engine to look for websites, Play Store to look for Android apps, and App Store to look for iOS apps. We defined a list of keywords (see Multimedia Appendix 1) in the English language for each eligibility criterion (ie, target group, scope, and chronic disease/condition) to be used alone or in combination (eg, target AND scope, target AND disease, scope AND disease, target AND scope AND disease). Each partner was responsible for translating the keywords into their national language.

### Selection Criteria

Resources identified through the search were screened for the eligibility criteria and, if eligible, assessed using a set of inclusion/exclusion and additional evaluation criteria (Textbox 1).

The resources were selected if they were available in the national languages of the participating countries (ie, Greek, Italian, Portuguese, Swedish). Researchers from Greece, Cyprus, and Portugal also searched and selected English resources, because in these countries there was a paucity of applications for carers and a large number of care workers speak English, thus could benefit from these resources. Apps were included only if they were free or available at a very low cost, defined as a price ≤€3. Websites were selected only if they were responsive for mobile devices (ie, they were designed to be optimized and easy to read on these devices). Responsiveness was assessed using the Google test tool [45].

Resources were evaluated for their update status, excluding resources that were clearly out-of-date. This assessment considered the content of the app or website, rather than a specific timeframe: For example, information about services and laws should be updated regularly, while an app about dietary habits may not necessarily be updated regularly, yet could still remain valid.

Usability was assessed with standardized tools. Apps were evaluated using the Mobile App Rating Scale (MARS) [46], a multidimensional scale developed to rate and measure the quality of health-related apps. The scale is composed of 23 items organized in 5 subscales (4 related to objective quality criteria and 1 for subjective quality), a classification section for descriptive purposes, and an optional set of app-specific items. The overall app quality score is calculated using the objective quality section, which is composed of 19 items, measuring whether the app is engaging, not boring, if it works properly, if its design is professional, and if the content is of high quality and capable of providing support. Each item is rated with a score ranging from 1 (ie, inadequate) to 5 (ie, excellent); some items can be evaluated as “not applicable.” The total score ranges from 1 (ie, very bad) to 5 (ie, very good), and the apps were included if they had a score ≥3. The usability of websites was evaluated with the System Usability Scale (SUS) [47], a simple and reliable tool for measuring the usability of a variety of products and services. The scale is composed of 10 items, rated from 1 (ie, strongly disagree) to 5 (ie, strongly agree). Total scores range from 0 to 100, and websites were included if they reached a score ≥68 [47].

In addition to these criteria, the resources were checked in terms of reliability and appropriateness of the content, data security, and privacy. These aspects, due to the wide range of resources evaluated, were not always applicable and contributed to the overall score definition, rather than producing an immediate decision of inclusion or exclusion.

Regarding reliability, health-related apps and websites were evaluated to verify whether they were certified, developed, or endorsed by relevant organizations in the field, whereas other types of resources were evaluated according to the relevance and utility for the target group. For example, we checked if health-related resources were endorsed or reviewed by established organizations (eg, Ministry of Health, medical associations, organizations like ‘Health on the Net’) or if they were included in repositories developed by European organizations (eg, myHealthApps). Regarding wellbeing and other resources, we verified if sufficient information about the developer was present or if the content was appropriate, for example, by checking if there were experts in the field that were involved in developing or revising the contents. Resources that were clearly not reliable (eg, no information about the developer, no clear content) were excluded. For-profit resources were excluded as not appropriate for the specific objectives of this study.

Data security and privacy were evaluated by checking if the app or website considered these issues and ensured their compliance. The resources were explored to verify the presence of elements such as appropriate terms of use; registration with the request to approve terms; explanation of how personal data are collected, treated, and stored; and request of permission to access to specific device features. In the case of apps, the informative page on the store was also scrutinized.

At the end of the evaluation, the reviewers assigned an overall score to each app and website, using a 5-star rating scale from 1 (ie, very bad) to 5 (ie, very good), taking into account the criteria and experience of using the resources, keeping in mind the target group of carers. This score was useful when multiple apps or websites had similar or same features, aims, and content, since it allowed us to identify the most suitable ones to be included in the app.

### Evaluation Process

The evaluation process was conducted in parallel in the 5 countries, following common procedures and tools. Specific guidelines were developed, including the list of keywords; definition of criteria and how to rate them; and standardized tools for the usability evaluation (ie, MARS and SUS), their explanation and scoring method, and related references. Regarding the MARS scale, we suggested to the evaluators to watch the video tutorial prepared by the authors of the scale as a training tool. Moreover, a standard spreadsheet was developed for the data extraction for each resource, with prefilled formulae for the calculation of the usability criteria. The guidelines and the spreadsheet were tested by two independent researchers and approved by all participating countries. In order to standardize the evaluation procedure and find consensus on each criterion, a pilot was conducted prior to the start of the selection process. In each country, two independent evaluators assessed at least two apps and two websites, comparing results and annotating divergent issues. An online meeting was organized to discuss the pilot results, and additional indications were defined to harmonize the review. Any difficulties encountered were discussed with a senior project researcher, who checked the procedure. Moreover, regular online meetings and discussions led to reaching consensus on the evaluation procedure.

Resources found through the online search were reviewed based on the criteria summarized in Textbox 1. Websites were evaluated by navigating across pages, while apps were reviewed first through the description page in the app store, second during the installation procedure, and finally by using the apps. At the end of the review, a list of resources was compiled, and a first classification was made, according to the resource scope.

**Criteria for the selection of the online resources.**

**Eligibility criteria**

- Target group: at least one group, preferably carers
  Carers
  Care recipients
  General public
- Scope: at least one
  Care planning and management
  Information and microlearning
  Communication and social inclusion
- Diseases/conditions: at least one
  Cardiovascular diseases
  Stroke
  Respiratory diseases
  Neurodegenerative diseases
  Cancer
  Musculoskeletal diseases
  Diabetes
  Mental illnesses
  Digestive diseases
  Sensory organ diseases
  Urinary incontinence

**Inclusion/exclusion criteria**

- Language: at least one national language; for Greece, Cyprus, and Portugal, English resources were also eligible
  Italian
  Greek
  Portuguese
  Swedish
  English
- Operating system and version (apps only): at least one
  Android
  iOS
- Price of apps or website subscriptions (apps only) ≤€3
- Responsive (websites only), as determined via assessment by the Google test tool [45]
- Update status: not being clearly out-of-date
  Apps: version and date
  Websites: date of the last update
- Usability
  Apps: Mobile App Rating Scale (MARS) quality score ≥3
  Websites: System Usability Scale (SUS) score ≥68

**Additional evaluation criteria**

- Reliability and appropriateness of the content: at least some information about reliability and appropriateness; not for-profit resources
  Health-related apps and websites: presence of endorsement by Health Ministry or other relevant organizations (eg, Health on the net) or included in European Directories (eg, myHealthApps Directory)
  Wellbeing and others: presence of sufficient information about developer or appropriateness of the content
- Data security and privacy, based on the content and purpose of the app or website
  Evaluation of security and privacy assured/considered (eg, terms of use, registration, information about data collection and storage)

### Data Extraction and Analysis

A standard spreadsheet was used to extract the data for the eligible resources. We retrieved a set of information for each resource, such as general information (ie, country, name, link, operating system available, and tested), eligibility criteria (ie, target, scope, disease), and inclusion/exclusion criteria (ie, language, price, update status, responsiveness, MARS quality score, SUS score). For included resources, we collected additional data, such as short description, keywords, reliability of the content, data security and privacy, name of the developer, registration needed (yes/no), expert rating, and any other relevant notes. For the apps, we added information on the need of an internet connection to use the app (yes/no), MARS subjective score, and user ratings. Descriptive statistics were used to summarize the main characteristics of the selected resources, by type (ie, apps and websites) and country. Data are expressed as frequencies or mean (SD). Data analysis was performed with the statistical software SPSS.

### Qualitative Study

After the selection of the resources, we conducted a qualitative study with informal carers through focus groups, a qualitative technique aimed to collect opinions, attitudes, and perceptions from a group of selected people [48]. Participants were asked to evaluate the appropriateness of resources and their classification. The list of resources was subsequently refined, and the process concluded with a list of resources classified by categories and subcategories [49].

### Focus Group Methods

We conducted 8 focus groups, 2 for each participating country (ie, Cyprus, Italy, Portugal, Sweden), except Greece. Because Greece and Cyprus share the same language, have a similar cultural background, and similar carer profiles, the results obtained in Cyprus can be generalized to Greece. The activities were carried out in parallel in all countries between March 2017 and June 2017. Common guidelines were defined and followed by all countries. These included the recruitment checklist, focus group guide, questionnaire for the participants, guidelines on how to conduct a focus group, guidelines for the analysis, spreadsheet for the collection of questionnaire data, and matrices to analyze and summarize the transcriptions.

Participants were selected according to the following inclusion criteria: being an adult informal carer of a frail adult or an older care recipient; providing at least 4 hours per week of support, excluding financial support or companionship [50]; caring for a care recipient who needed support in activities of daily living (eg, mobility) or had a noncommunicable disease; being the primary carer of the care recipient; owning a smartphone or tablet; and having at least medium digital skills (ie, being able to use apps and surf the web on his or her own). These criteria were chosen to select carers with a high burden of care, representing the target group that could most benefit from the use of mobile resources. Moreover, we needed carers with at least medium-level digital skills so that they would have some prior knowledge and experiences with using mobile devices, which are the focus of the study, and would be able to provide their opinions on the points raised during the focus group.

The focus group guide included an introduction to present the project and objectives of the focus group, followed by 4 sections: (1) use of smartphone or tablet and apps; (2) evaluation of selected resources; (3) the Apps4carers visual prototype evaluation (including classification of the selected resources); and (4) training courses and material. The results presented here refer to the first and second sections and the topic of classification to the third one. The evaluation of the resources (section 2) was carried out by presenting to the carers the kind of resources included in each scope, explaining how these resources could support carers’ tasks, and providing at least 2 or 3 examples of apps or websites for each scope, by screenshots (ie, storyboard) or navigating the apps or websites in real-time (ie, walkthrough). To avoid an extensive and potentially burdensome discussion with participants, two focus groups were organized in each country, each one addressing different categories of resources and assuring, in this way, that all categories were covered. Regarding the classification of the resources, we presented and explained the classification used and asked carers to evaluate it in terms of clarity of both the names of the categories and resources included in each one.

Recruitment of potential focus group participants was carried out through a checklist and via phone calls or face-to-face contact with participants, mainly through carers’ associations or health or social care services. In each country, suitable locations for the focus groups were selected, considering comfort and accessibility for participant carers. Immediately prior to the session, participants were requested to sign an informed consent form explaining the aims of the project and procedures. A short sociodemographic background questionnaire was also administered to participants, in order to gather the main sociodemographic characteristics of both the carers (ie, age, gender, marital status, education, working status, children, relation with care recipient) and the care recipients (ie, age, sex, duration of care, hours of care per week, living conditions, level of dependency, financial support or allowances, presence of a private care assistant).

Focus groups were conducted by qualified personnel (eg, social researchers, psychologists) and involved a moderator or facilitator leading the focus group and an assistant moderator or observer listening to the focus group's discussion and taking detailed notes. Each discussion, with the participants’ permission, was audio-recorded and transcribed verbatim. Each focus group lasted about 2 hours.

### Focus Group Analysis

Due to the different languages, focus groups were analyzed by each country project team separately using a common template, and the results were merged to produce a common report. The qualitative data were analyzed manually adopting the framework analysis technique [48,51], a case and theme-based approach that reduces data through summarization and synthesis using a matrix. This method helped to order data, allowing the researchers to analyze them both by case (ie, the carer’s point of view) and by theme. Data management and interpretation are sequential: it starts deductively from the aims and objectives of the study but reflects the original observation of the people (ie, it is “grounded” and “inducted”). Initially, focus group transcriptions were read multiple times for the researchers to become familiar with them. A set of a priori themes were defined based on the objectives of the study and the focus group guide, to be further integrated with additional themes, if necessary. A standard matrix was used to summarize and analyze data of each theme, where rows represented participants and columns the subthemes. Each country was asked to provide a summary of the results for each section and subthemes, including relevant quotations, if any.

## Results

### Selection of Mobile Resources

**Figure 1:**
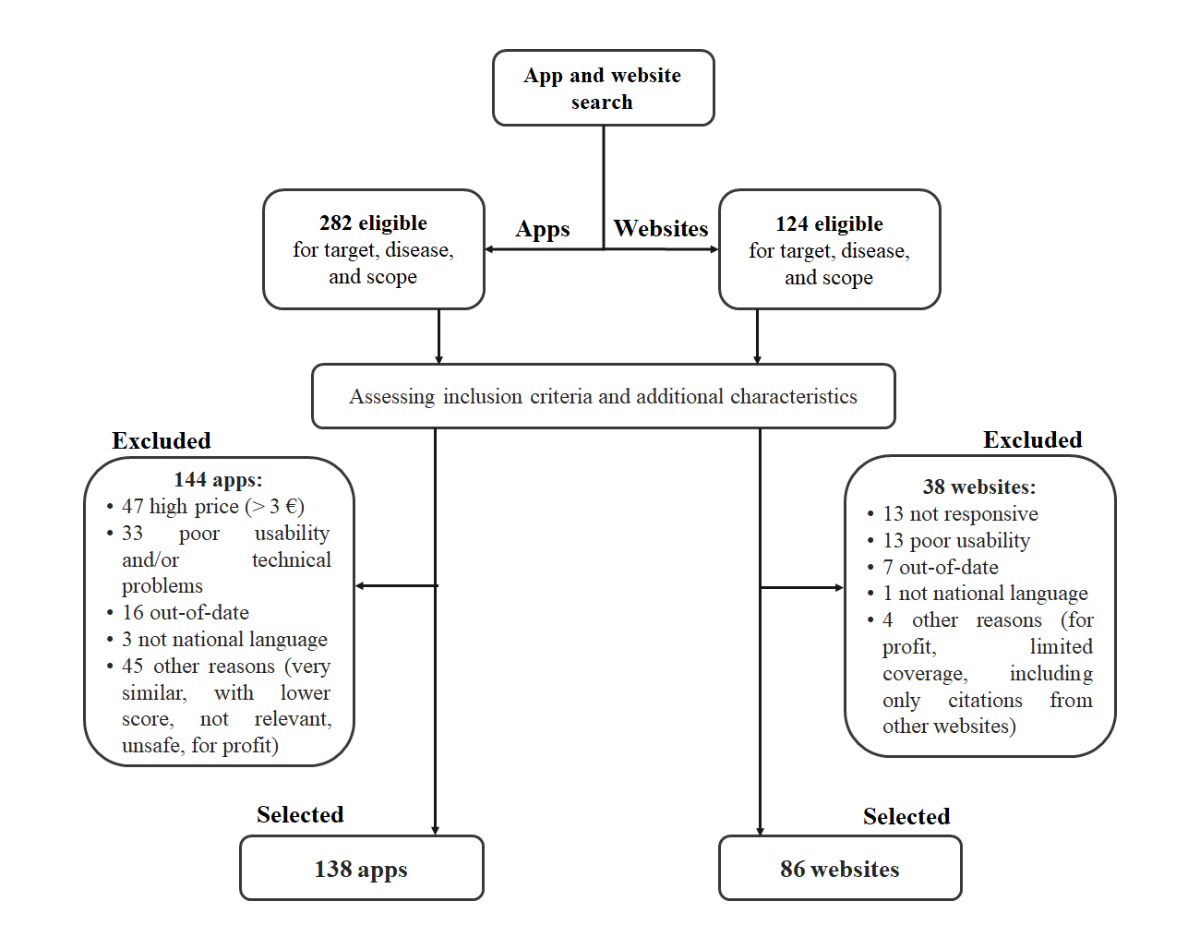
Flowchart of the selection process.

The search strategy retrieved 406 resources eligible for target group, disease/condition, and scope (Figure 1), of which 282 were apps and 124 were websites. Assessment of the inclusion and exclusion criteria and additional evaluation criteria led to the exclusion of 182 resources (144 apps and 38 websites) and the selection of 224 resources (ie, 138 apps and 86 websites; see Multimedia Appendix 2) [49]. The main reasons for exclusion of the apps were price, usability or technical issues, and update status, whereas websites were excluded primarily because of a lack of responsiveness, usability, and update status.

### Characteristics of Selected Resources

The selected resources were classified into 3 categories, according to their scope. In the first category, “Care plan and management,” we included resources useful for carers in the organization and planning of daily and long-term care tasks, such as keeping track of therapy or symptoms of a disease and sharing information with other family member or with a health care professional. The second category, called “Information and microlearning,” collected resources providing information about the diseases (eg, symptoms, therapies) and about any public or private services that offer support to the care recipients and their carers. Finally, the category “Communication and social inclusion” compiled the resources providing support, advice, or sharing of information and experiences with other carers. Each resource was allowed to be related to more than one category.

The main characteristics of the selected resources are shown in Table 1.

The number of apps was quite homogeneous across countries, whereas websites varied from 5 in Portugal to 41 in Sweden. Among the selected apps, 35 were in the English language (of which 20 were in Greece/Cyprus and 15 were in Portugal). Only 17.0% (38/224) of the resources were devoted specifically to carers, whereas 22.3% (50/224) of all resources and 14.5% (20/138) and 35% (30/86) of apps and websites, respectively, addressed both carers and care recipients. As expected, apps were mainly related to issues of care planning and management, while websites provided mainly information and microlearning. Half of the resources (109/224, 48.7%) were disease-specific, specifically addressing one or more diseases/conditions of the care recipient. In most cases, these were represented (data not shown) by neurological diseases (eg, Alzheimer’s disease) for both apps (22/138, 15.9%) and websites (32/86, 37%); stroke (12/138, 8.7% and 12/86, 14%, respectively), followed by cardiovascular diseases (11/138, 8.0%) and diabetes (8/138, 5.8%) for apps; and musculoskeletal diseases (10/86, 12%) and cancer (8/86, 9%) for websites. The remaining resources not specifically addressing one disease/condition included information and utilities on a variety of themes, such as finding a pharmacy, explaining social services or legal aspects, reminders for therapies and medications, utilities for the older person, or emergency numbers.

The majority of the resources (153/224, 68.3%) were evaluated as reliable and with appropriate content, while we were able to verify the assurance of data security and privacy for only 77 resources (77/224, 34.4%). However, regarding this issue, it should be noted that a high number of resources only provided information, which in general does not imply any request or use of personal data from the user. As for the mode of use, 59.4% (82/138) of the apps required registration, and 50.7% (70/138) needed an internet connection. Expert ratings reached a mean score of 3.7 (SD 0.7) on a scale from 1 to 5. User ratings, purely related to the apps, were slightly higher, at a mean score of 4.0 (SD 0.6).

**Table 1:** Characteristics of the selected resources.

		Apps (n=138), n (%)	Websites (n=86), n (%)	Total (n=224), n (%)
**Country**				
	Greece and Cyprus^a^	35 (25.4)	16 (18.6)	51 (22.8)
	Italy	42 (30.4)	24 (27.9)	66 (29.5)
	Portugal^b^	33 (23.9)	5 (5.8)	38 (17.0)
	Sweden	28 (20.3)	41 (47.7)	69 (30.8)
**Operating system**				
	Android	22 (15.9)	N/A^c^	N/A
	iOS	25 (18.1)	N/A	N/A
	Both	91 (65.9)	N/A	N/A
**Target group**				
	Carers	25 (18.1)	13 (15.1)	38 (17.0)
	Carers and care recipients	20 (14.5)	30 (34.9)	50 (22.3)
	Care recipients	17 (12.3)	13 (15.1)	30 (13.4)
	General public	76 (55.1)	30 (34.9)	106 (47.3)
**Scope^d^**				
	Care plan and management	72 (52.2)	20 (23.3)	92 (41.1)
	Information and microlearning	80 (58.0)	73 (84.9)	153 (68.3)
	Communication and social inclusion	29 (21.0)	31 (36.0)	60 (26.8)
Disease-specific (yes)		58 (42.0)	51 (59.3)	109 (48.7)
**Reliability/appropriateness of the contents**				
	Not reliable	1 (0.7)	0 (0.0)	1 (0.4)
	Reliable	91 (65.9)	62 (72.1)	153 (68.3)
	Not assessed	46 (33.3)	24 (27.9)	70 (31.3)
**Data security and privacy**				
	Not assured	9 (6.5)	5 (5.8)	14 (6.3)
	Assured	50 (36.2)	27 (31.4)	77 (34.4)
	Not assessed or not relevant	79 (57.2)	54 (62.8)	133 (59.4)
Registration needed (yes)		82 (59.4)	10 (11.6)	92 (41.1)
Connection needed (yes)		70 (50.7)	N/A	N/A
Usability^e,f^		138, 3.6 (0.4)	81, 81.5 (8.7)	N/A
User rating (1-5)^f^		117, 4 (0.6)	N/A	N/A
Expert rating (1-5)^f^		138, 3.7 (0.7)	86, 3.8 (0.7)	224, 3.7 (0.7)

^a^20 apps were in the English language.

^b^15 apps were in the English language.

^c^N/A: not applicable.

^d^Each resource could have multiple scopes.

^e^Usability was assessed using the Mobile App Rating Scale for the apps and the System Usability Scale for the websites.

^f^Reported as n, mean (SD).

### Focus Group Results

Focus groups involved 47 carers in 4 countries (ie, Cyprus, Italy, Portugal, Sweden) and aimed to evaluate the selected resources and their classification. Overall, enrolled participants had a mean age of 55 years (SD 12.0) years, were mainly female (32/47, 68%), were married (32/47, 68%), had a medium-high level of education (39/47, 83%), were employed (26/47, 55%), and had at least one child (33/47, 70%). They were providing care primarily to a parent (29/47, 62%) or a spouse/partner (12/47, 26%). About half of the carers (19/42, 45%) provided assistance for 2-4 years, while 36% (15/42) provided assistance for longer than 5 years. More than 20 hours per week of care was provided by 48% (20/42) of participants; among these participants, 10 people were providing full-time care (10/42, 23.8%). Regarding the living situation, 17 carers (17/46, 37.0%) lived with the care recipient, while 18 (18/46, 39%) lived within walking distance. Care recipients had a mean age of 77 years (SD 9 years) and were, to a large extent, slightly or moderately dependent (32/46, 69%), while 28% (13/46) were severely dependent. Only 38% (17/45) received financial support or an allowance from the state or public organizations, and 34% (15/44) were paying for a private care assistant who provided part-time or full-time support.

The focus group results are presented in Table 2, according to themes, subthemes, and country. Three a priori themes are reported, including the use of devices and resources, evaluation of resources, and classification of resources.

Regarding the use of mobile devices (ie, smartphones, tablet), all participants owned a smartphone or a tablet, but the majority reported limited knowledge of smartphone features and functions, stating that they used their devices only for basic activities. Only the Swedish group included some expert users who were quite confident in using apps, for both daily activities and caring duties (eg, searching for health information, setting reminders, using locators). Italian and Portuguese carers reported usually asking for the help of their relatives to download, install, and use apps. In relation to resources (ie, apps, websites) dedicated to carers, almost all of the participants had no previous knowledge of resources specifically targeting carers. Most used apps and websites to support daily tasks (eg, communication, navigation, banking, social networking, games) or to find the information they needed (eg, travels, news, online shopping).

The second theme was the evaluation of the resources selected in each country, in terms of previous knowledge, interest, potential benefits, barriers, and perspective about resources in the English language. Overall, participants expressed a major interest in the resources presented during the focus groups, considering them useful tools for care provision and assistance. Some participants took note of the names or installed them immediately after the end of the discussion. Some remarks were raised by several participants, who reported that some selected resources were not always relevant for experienced carers, yet they could be deemed useful for novice carers. Another issue raised was that the resources could be useful but they do not solve practical issues of service availabilities, do not replace the need for direct or face-to-face contact with health and social care professionals, or they lack a “local” perspective.

Most of the participants did not know any of the apps or websites that were presented; however, they all seemed highly interested in their functions and potential benefits. The perceptions of the carers about the examples of resources presented were generally positive. They reported that the apps and websites seemed easy to use and intuitive. Moreover, the carers appreciated the resources for the following aspects: the relevant information provided (eg, disease explanation, tips and guidelines for specific actions or events), presence of multiple functions (eg, drug reminder and therapy management instead of a simple alarm clock), interactive approach (eg, guided exercises, personalized plans), entertainment aspect (eg, digital photo album), and possibility to share information with health and social care professionals or other informal carers. The carers reported that the availability of these resources could make them feel safe because, in some cases, these resources could be lifesaving (eg, emergency events, locators for people affected by dementia), could reduce anxiety about tasks to be performed (eg, checking drug therapy), and could facilitate care management (eg, recipes for specific conditions). An additional aspect raised by the Italian carers was the potential benefit of the use of these resources by paid carers, in order to improve the quality of care provided.

When asked about obstacles and barriers that could prevent carers from using these resources, participants indicated as their main concern the low level of digital skills of adult carers, in particular among older carers. For example, some apps were evaluated as being too complex, requiring more competency to be used, while others required the care recipients to have and use a smartphone, which could be problematic due to their low digital skills or even their fear of using new technologies. Some participants reported that the resources designed for care recipients would be more suitable for carers instead. When talking about health-related apps, some Swedish and Cypriot users raised the issue of reliability, explaining that what prevented them from using online resources is the uncertainty of the accuracy and reliability of the information provided in the apps.

With the exception of the Greek and Cypriot users, participants from other countries were not interested in apps available in languages other than their national language (ie, English language). However, multilingual resources were considered to be potentially helpful for privately paid care assistants, in most cases with a migrant background, who may have limited knowledge of the language of their host country.

As for the classification of resources, participants had some difficulties in understanding which kind of resources were included in the 3 categories (ie, care plan and management, information and microlearning, communication and social inclusion). The main concerns raised were related to the words selected to represent the categories (in each national language), which were evaluated as too general in some cases or too difficult to understand what they consisted of in other cases. The moderators elicited the provision of suggestions to improve the classification and labels associated with each category, to inform the subsequent development of the project’s app. Main suggestions referred to the use of simple and widely understandable words, possibility to receive a brief explanation of what is included in each category, and use of graphic elements.

**Table 2:** Focus group results.

Theme and subthemes		Results
**Use of devices and resources**		
	Use of smartphone or tablet	All owned smartphones or tablets (IT^a^, CY^b^, SE^c^), some also computers (SE); most owned smartphones/tablets, some only personal computers (PT^d^)
	Digital skills	Half needed support to download and install apps (IT, SE); only some had difficulty with finding and downloading the apps (CY); only half were familiar with the “app concept” (PT)
	App use and opinions	Daily tasks, communication, entertainment, and searching for information (IT, CY); basic functions (PT); communication, entertainment, paying bills, reading news; and searching for health information on the web (SE)
**Evaluation of resources**		
	Previous knowledge of the resources	None knew of the presented apps/websites (IT, PT); most did not know the presented apps/websites, while some were familiar with similar resources (CY); some participants had heard about some app or even had it on their smartphones, while some did not know of any resources (SE)
	Interest	Great interest towards the resources (IT, CY, PT); generally interested, while experienced carers found the selected resources not always relevant for them (SE)
	Benefits	Considered helpful for care provision and assistance (IT, PT, CY) and for obtaining reliable information (CY); not many were mentioned, while participants felt their caring situation was too complex for an app to be helpful (SE)
	Barriers	Low digital skills of carers (IT, PT) and care recipients (IT); usability and reliability issues, poor Greek translations (CY); problems concerning the trustworthiness of apps (SE)
	English language	They would not use any app in English (IT, PT, SE); English resources could be useful for immigrants and migrant paid assistants (CY)
Classification		Categories and labels were not clear or easy to understand (IT, CY, PT, SE)

^a^IT: Italy.

^b^CY: Cyprus.

^c^SE: Sweden.

^d^PT: Portugal.

### Final Classification of the Resources

The focus group results were used to “validate” the list of selected resources, confirming their usefulness for the carers and the need to improve awareness about the resources among carers and their organizations. However, the draft classification devised using the scope of the resources was not widely understood by the carers. Although the focus group participants were not able to provide specific suggestions for improvements, they provided valuable information about their needs and their perceptions of the resources. These elements, together with an in-depth analysis of the resources selected, supported the researchers in the definition of a new, more accessible, and coherent classification. First, we decided to highlight, by creating a separate category, the resources specifically targeted to carer wellbeing (eg, relaxation techniques). The second step was to identify, for each resource, the main feature, functionality, or aim and link it to a major carer need, to facilitate the carers, especially novices, to find the appropriate resources. For this purpose, the analysis of the resources led to the selection of three needs: management of the health status of the care recipient, connection with individuals or organizations able to support the carers in their tasks, and the need to have practical tools to overcome barriers and problems while caring (eg, communication). These considerations led to the final classification presented in Table 3, including 4 categories and, for each, a classification in subcategories, with examples provided for descriptive purposes. The first category was named “Carer wellbeing” and included resources supporting carers in dealing with stress management, such as relaxation techniques and mindfulness. The second one, called “Managing health and diseases of the care recipient,” comprised not only the resources related to care planning but also those providing information about the diseases/conditions of the care recipient. Services, helplines, and peer support were grouped under the label “Useful contacts,” whereas utilities and tools potentially helpful in the care of older people have been placed under the category “Technologies for eldercare.” In this classification, each resource was allocated to only one category. It was also decided that the online library would include a fifth category comprising all the resources with the possibility for the carers to search among them by using filters and keywords. This option aimed to make the app more accessible and suitable also for skilled carers.

**Table 3:** Final taxonomy of the selected resources.

Category and subcategories		Kind of resources included (examples)
Carer wellbeing		Tools devoted to carers (eg, relaxation, stress management)
**Managing health and diseases of the care recipient**		
	Diseases and health-related issues	Information, advice and tips to manage symptoms and problems, rehabilitation tools (eg, cognitive training), assessment and tracking of parameters, sharing information among carers and professionals, prevention
	Medications	Information, reminders and management
	Nutrition and diet	Information and advice, tracking systems
**Useful contacts**		
	Services	Services: information, research, and location (eg, pharmacies); associations: information and events; legal and financial information and advice
	Help and information	Helplines; emergencies: direct link, calls; emergencies: information; peer support and expert advice (eg, Facebook groups and fora)
Technologies for elder care		Resources for disabled people (eg, interfaces, assistive devices)

## Discussion

This study aimed to review and select available mobile resources supporting informal carers of frail adults or older people in 5 European countries. The combined use of standardized tools, real tests, and focus groups provided a comprehensive review of the actual “state-of-the-art” status of mobile resources for carers. We found few resources dedicated to carers, and we covered the missing aspects with alternatives targeting care recipients or the general population. We also found that there are some underdeveloped areas and some specific features requiring attention and additional effort by the developers of apps and websites. Finally, the focus groups confirmed the usefulness of the selected resources, underlined the main barriers to their use, and led to the definition of a new taxonomy.

In greater detail, the main result emerging from this study concerned the paucity of apps and websites specifically developed for informal carers. We identified only 25 apps and 13 websites addressing this target group, aiming to provide guides to stress management; information, advice, and tips to provide care for people with different diseases (eg, dementia, Parkinson’s disease, stroke); or events and services for carers. These results are in line with a recent review conducted in the United States [24], which found only 44 apps for carers of older adults. The lack of adequate resources able to support carers is an indirect sign of the largely unrecognized role they play in everyday care for older people in our aging societies. Informal carers do provide a vast amount of caring activities and thus enhance the sustainability of welfare systems [52,53], in a context where the need for long-term care is increasing [54] and the number of people potentially available to provide care is declining [55]. At the same time, there is evidence that informal carers are at a higher risk of developing carer burden and psychiatric morbidity [56], meaning that they also need support to deal with their wellbeing and caring situation. One possible solution to this problem is to provide valuable ICT-based support tools to carers, so they can provide good quality care and receive support for themselves [19]. The potential usefulness and beneficial impact of these tools were confirmed by the carers involved in the focus groups. In the majority of cases, they were not aware of these resources but, once presented, they understood their potential and were willing to use them for their caring activities.

The needs and preferences of carers vary considerably and differ by personal characteristics of both the carer and the care recipient, the kind of relationship between them, and the stage of the caring cycle [28,57-59]. For example, when assuming this role, carers have to acquire all useful information and training related to the disease/condition affecting the care recipient. Then, they often search for communication and support services, both formal and informal. Finally, they also need support from peers or care professionals to manage the stress and burden caused by caring activities, often accumulating over time. The carers’ opinions collected during the focus groups confirmed these differential needs and the necessity to identify relevant online resources able to address them. In our review, we found apps and websites covering all these aspects, even if some areas were more developed compared to others. The majority of the selected resources provided information, followed by different apps for monitoring disease(s) and medicines. In the area of peer support, we found a few, but interesting, resources (eg, Facebook groups), which are likely to increase in the future thanks to the diffusion of social networks. These resources represent a source of information exchange and support for carers and a way to increase the effectiveness of their role and reduce the stress associated with the caring activities [60].

We found a paucity of resources aiming specifically to manage stress and burden, such as guided exercises of relaxation techniques or meditation. This is a promising sector, and a recent review [61] underlined the positive effects of online mindfulness-based interventions on mental health.

Although the area of information revealed the highest number of resources, there is a need to further address issues of reliability of the content [29,30] and usability [26,62]. In many cases, the absence of indications of endorsement or revision by experts or by devoted bodies hampered even the evaluator’s assessment of the reliability of the content for both apps and websites. The focus group findings confirmed that these issues often prevented carers from using the resources. Evaluation and trust of web-based health information by users is related to eHealth literacy and mHealth literacy and is influenced by carers’ characteristics such as socioeconomic position, education, and employment [31,32,63]. Therefore, there is a need to train carers on how to evaluate the quality and accuracy of online health information and to increase the use of certified web-based resources that are easily recognizable. Second, a consistent number of resources were excluded because of poor usability and technical problems. Although we evaluated only free or very cheap resources, in many cases, paid apps were just “premium” versions of free apps, where more content or functionality are provided to the user with the same design. Moreover, when considering websites, many were excluded due to the lack of a “mobile” version. Importance of usability was also confirmed by the users, who reported the risk of not using or ceasing to use apps if they are too complex. This was the case for the classification used for the selected resources, which failed to be understood by the carers and required a complete revision.

Finally, the focus group results highlighted that carers having different characteristics and from different European contexts shared the same opinions and perspectives on the use and usefulness of mobile resources. This provides evidence of the potential impact and transferability of this initiative in other European countries.

Despite the original findings presented so far, this study has some limitations, too. First, although the review and selection processes used a systematic approach, the search could have excluded some relevant resources, due to the functionality of the tools used for the search or due to the translation of the keywords in the national languages. Second, we restricted our search to the major diseases/conditions associated with a high burden of care. Although this could have missed some resources, we do consider that we covered the majority of the needs of the target group considered. Third, the evaluation of the resources in terms of update status, reliability/appropriateness of the content, and data security and privacy was carried out by the researchers following a set of general indications, thus not assuring an objective rating. Finally, given the relatively short timeframe of our project, it was not feasible for the selection of the resources to also undergo a review by external experts in all countries, which might have ensured additional validation of the obtained results.

Notwithstanding these limitations, this study allows us to state that mobile resources are potentially valuable tools for carers, as they can be used anytime and anywhere to search for information, contact persons, and learn something new and relevant about the caregiving role [64]. Moreover, these tools could also have a positive impact in terms of sustainability of the welfare system and overall cost savings [59]. This study confirmed that, although mobile resources such as apps and websites could support carers in their tasks, more effort should be spent in creating a greater awareness about these resources and in developing good-quality resources specifically targeted to carers, by addressing their needs and preferences [18]. To reach these objectives, apps and websites need to be developed and co-designed together with the end users while enhancing the acquisition of digital skills needed to use these tools. We further exploited the results of this study in the subsequent phases of the project, by developing an online library of the selected resources [33] and training materials to support carers wishing to learn how to use these tools. The main future challenge, for both research and market fields, will remain the understanding of how to best fill the gap between the potential benefits for carers of using ICTs and their real use of these resources.

## Appendix

Multimedia Appendix 1 List of keywords used for searching the resources.

Multimedia Appendix 2 List of selected resources.

## Abbreviations

eHealth: electronic health.
ICT: information and communication technology.
MARS: Mobile App Rating Scale.
mHealth: mobile health.
SUS: System Usability Scale.
