# Supplementary material for: Review and Selection of Online Resources for Carers of Frail Adults or Older People in Five European Countries: Mixed-Methods Study
Source: JMIR Mhealth Uhealth. 2020 Jun 17;8(6):e14618. doi: 10.2196/14618 (PMC7330736; doi:10.2196/14618)
Supplement: Multimedia Appendix 1 [file mhealth_v8i6e14618_app1.docx]

# **Multimedia appendix 1.** List of keywords used for searching the resources.

**Keywords per SCOPE (**uppercase words are relevant for more than one category)**:**

| Category | Sub-category | Keywords |
| --- | --- | --- |
| **Care plan and management** | Tracking System/Devices | Health report/data/assessment/monitoring/information, health data manager/management, symptoms checker, monitoring, pain monitoring/tracker, video monitoring, sleep monitoring, cognition measurement, heart monitoring, mood assessment, memory, motion, mobility assessment, blood sugar/ glucose, pressure, cholesterol, localization, RISK FACTORS FOR FALL, REPORT, HYPERTENSION, gps, family sharing and location screen, balance assessment, SELF-CHECK |
|  | Care coordination | Daily task management/manager/reminder/organizer, notification, residential setting, care information, CARE PLAN/MANAGEMENT, appointments, ALERT/ALARM, domestic care work/home help, MEDICINES PURCHASE, CO-MORBIDITY |
|  | Medication Management | Medication, medication monitoring/reminder/management, pills/medicine/drug, palliative care, CARE PLAN/MANAGEMENT, medical events, ALERT/ALARM, ALARM REPORT, ANTIPSYCHOTIC MEDICATION, MEDICINES PURCHASE, side effects |
|  | Everyday life tasks | DIET/DIETARY GUIDANCE, personal care, reminder |
| **Information and micro-learning** | Medical and Health information | Disease, disease information, behavioural changes management, HYPERTENSION, autism, aphasia, disabilities, comorbidity, ANTIPSYCHOTIC MEDICATION, RISK FACTORS FOR FALL, LEARNING, ADVICE/ tips, tutorial, information, care, care information, medicine information, healthy ageing, DIET/DIETARY GUIDANCE, quiz/questionnaire, CO-MORBIDITY |
|  | Stress management and wellbeing | Coping strategies, coping with depression, burden level, relax, SUPPORT/GUIDANCE/ADVICE, stress management, COUNSELLING, autogenous training, mindfulness, LEARNING, SELF-CHECK |
|  | Public/Private services and local initiatives | Search engine, services/facilities, on-duty pharmacies, pharmacy, pharmacy locator, doctors, hospitals, association, scientific/national/non-profit association, home healthcare services, care professionals, healthcare information/providers, access, ramps, silver alert services, emergency, emergency tools/numbers, assistance request, useful contacts, respite care |
|  | Legal and financial information and support | Benefits, allowances, advocacy, legal information/advice |
| **Communication and social inclusion** | Peer support resources | Virtual communities, SUPPORT/GUIDANCE/ADVICE, network/network of contacts, social networks, COUNSELLING, peer-support, COMMUNICATION/ conversation, blog, hotline |
|  | Assistive devices | Readability, sign language, people with hearing impairments, people with disabilities, COMMUNICATION, aphasia, communication helper/facilitator |

**Keywords for CHRONIC DISEASES/CONDITIONS:**

| Chronic diseases/conditions | Keywords |
| --- | --- |
| **Cardiovascular diseases** | Coronary heart disease, Chronic heart failure, Congestive heart failure, Angina pectoris, Chest pain, Myocardial Infarction, Heart Attack, Hypertension, Atherosclerosis, Heart Block, Arrhythmia. |
| **Stroke** | Stroke, Cerebrovascular Disease, Ischemic Stroke, Hemorrhagic Stroke, Transient Ischemic Attack, Vascular Dementia. |
| **Respiratory diseases** | Respiratory Impairment, Lung Disease, Pneumonia, Chronic obstructive pulmonary disease, COPD, Chronic lower respiratory disease,  Chronic respiratory failure, Asthma, Chronic Bronchitis. |
| **Neurodegenerative diseases** | Mild Cognitive Impairment, Alzheimer's Disease, Dementia, Parkinson’s disease, Multiple Sclerosis, Huntington Disease. |
| **Cancer** | Cancer, Tumor, Malignancy, Neoplasm + body location/system involved. |
| **Musculoskeletal diseases** | Bone disease, Fractures, Hip fracture, Osteoporosis, Osteoarthritis, Rheumatoid arthritis, Back pain, Falls prevention, Falls Risk, Balance loss, Gait changes, Chronic musculoskeletal pain. |
| **Diabetes** | type 1/type 2 Diabetes, Pre-diabetes, Hypoglycemia, Hyperglycemia, Hypercholesterolemia, Glycaemia,  Glycemic control, Lipid lowering. |
| **Mental Disorders** | Mental Health**,** Major Depressive Disorder, Depression, Mood Disorder, Schizophrenia, Anxiety. |
| **Digestive diseases** | Dysphagia, Swallowing disorder, reduced absorption of nutrients, Constipation, Eduntulism, Diverticular Disease, Ulcers, Polyps, Gastroesophageal reflux disease. |
| **Sense organ diseases** | Sensory loss, Visual impairment, Vision disorder, Eye disease, Sight loss**,** Hearing impairment, Hearing loss. |
| **Urinary Incontinence** | Functional Incontinence, Iatrogenic Incontinence, Stress incontinence, Urge, Overflow incontinence. |

**Keywords for TARGET GROUP:**

Carers, caregivers, elderly, older adults, senior citizens, dependent individuals, dependent care recipients, care recipients, disable individuals, people with dementia, people with memory disorders.
