# Supplementary material for: Review and Selection of Online Resources for Carers of Frail Adults or Older People in Five European Countries: Mixed-Methods Study
Source: JMIR Mhealth Uhealth. 2020 Jun 17;8(6):e14618. doi: 10.2196/14618 (PMC7330736; doi:10.2196/14618)
Supplement: Multimedia Appendix 2 [file mhealth_v8i6e14618_app2.pdf]

Multimedia appendix 2. List of selected resources.

Applications

Country	Name	Link	Target	Category	Disease/ condition	Description	Op. system	Reliability	Privacy	Registration	Connection	MARS	Score
**IT01**	Numeri Utili	https://play.google.com/store/apps/details?id=it.android.numeri&hl=it	General public	CONTACTS	Not disease-specific	This App provides the most important Italian national numbers grouped by section: emergency numbers, public services, etc. It discriminates the free phone numbers and the paid ones.	Android	-	Yes	No	No	3	3
**IT02**	iFarmacie di turno	https://itunes.apple.com/it/app/ifarmaciediturno/id960636682?mt=8	General public	CONTACTS	Not disease-specific	This App gives information on finding opened pharmacies.	iOS	-	Yes	No	Yes	3	3
**IT03**	Farmavie	https://play.google.com/store/apps/details?id=it.wikipharm.farmavie&hl=it	General public	CONTACTS	Not disease-specific	Pharmacy finder allowing users to search for pharmacies by name, by address, or using the GPS locator to list pharmacies nearby.	Android, iOS	Yes	-	Yes	Yes	4	3
**IT04**	PharmAround	https://play.google.com/store/apps/details?id=com.develon.pharmaround	General public	CONTACTS	Not disease-specific	This App gives information on finding opened pharmacies.	Android, iOS	-	-	No	Yes	3	4

Country	Name	Link	Target	Category	Disease/ condition	Description	Op. system	Reliability	Privacy	Registration	Connection	MARS	Score
**IT05**	Iperteso? Controlla anche l'uricemia	https://play.google.com/store/apps/details?id=it.euromultimedia.iperuricemia&rdid=it.euromultimedia.iperuricemia	General public	HEALTH	Cardiovascular	This app includes information about hyperuricemia and helps to manage this condition. It was designed and realized by the Italian Society of Arterial Hypertension (SIIA).	Android, iOS	Yes	Yes	No	No	4	4
**IT06**	La mia pressione del sangue	https://play.google.com/store/apps/details?id=com.mengtaoye.bloodpressure&rdid=com.mengtaoye.bloodpressure	General public	HEALTH	Cardiovascular	This app allows easily monitoring blood pressure and heart rate. It creates charts automatically, allowing to export and send data via e-mail.	Android	-	-	No	No	4	3
**IT07**	Promemoria per farmaci con allarme e calendario	https://itunes.apple.com/it/app/promemoria-per-farmaci-con-allarme-e-calendario/id816347839?mt=8	Carers & care recipients	HEALTH	Not disease-specific	Pill reminder with many functions: it allows creating reminders, to track the remaining quantity of each medication, get refill alerts, marking medications as "taken not taken", and adding notes for medical appointments. It allows planning the therapy for multiple users.	iOS	-	-	Yes	No	4	4
**IT08**	App Dieta Mediterranea Mdiet	https://play.google.com/store/apps/details?id=it.milkmaid.mdiet&rdid=it.milkmaid.mdiet	General public	HEALTH	Not disease-specific	This app includes information about foods and recipes typical of the Mediterranean diet, and allows creating a daily food diary.	Android	Yes	-	Yes	No	4	4

Country	Name	Link	Target	Category	Disease/ condition	Description	Op. system	Reliability	Privacy	Registration	Connection	MARS	Score
IT09	ESH CARE	https://play.google.com/store/apps/details?id=it.youco.siia&hl=it	Care recipients	HEALTH	Cardiovascular, Stroke	Hypertension app validated and supported by the European Society of Hypertension. It allows keeping track of blood pressure values, and sharing them with medical staff by email. Additionally, the app has a handy medicines reminder, and includes practical hints to keep hypertension under control.	Android, iOS	Yes	Yes	No	Yes	4	4
IT10	ICTUS3R	https://play.google.com/store/apps/details?id=com.ictus3r.app	General public	HEALTH	Cardiovascular, Stroke	ICTUS3R gives information on how to recognize signs of a stroke react correctly and reduce the risk.	Android, iOS	-	Yes	Yes	Yes	4	4
IT11	Ipertensione	https://play.google.com/store/apps/details?id=com.momentomedico.siia	Carers & care recipients	HEALTH	Cardiovascular, Stroke	This app provides detailed information on hypertension. It gives advice on how to keep a healthy lifestyle and reduce the cardiovascular risk. The user can record and monitor personal health data.	Android, iOS	Yes	Yes	No	No	4	4
IT12	Indice glicemico e carico IG	https://play.google.com/store/apps/details?id=com.creamsoft.mygi&hl=it	General public	HEALTH	Diabetes	This app allows to easily searching for the glycaemic index and glycaemic load of various foods, and their carbohydrate content.	Android, iOS	Yes	-	Yes	No	4	4
IT13	Indice glicemico e carico IG - premium	https://play.google.com/store/apps/details?id=com.creamsoft.mygi&hl=it	General public	HEALTH	Diabetes	This app contains a premium version. As the basic version, it allows to easily searching for the glycaemic index and glycaemic load of various foods, and their carbohydrate content. In addition, the premium version allows keeping a food diary, and to create and export charts.	Android, iOS	Yes	-	Yes	No	4	4

Country	Name	Link	Target	Category	Disease/ condition	Description	Op. system	Reliability	Privacy	Registration	Connection	MARS	Score
**IT14**	Glimp	https://play.google.com/store/apps/details?id=it.ct.glicemia&rdid=it.ct.glicemia	General public	HEALTH	Diabetes	This app records glucose levels in the blood, the units of insulin taken, the carbohydrate consumed, and the sport activity. In case, it can be connected with Dropbox to remote monitoring the results, and is compatible with Abbott sensors.	Android	-	-	Yes	No	3	3
**IT15**	MyTherapy Promemoria per pastiglie	https://play.google.com/store/apps/details?id=eu.smartpatient.mytherapy&rdid=eu.smartpatient.mytherapy	Carers & care recipients	HEALTH	Not disease-specific	This app helps managing therapies, tracking health conditions, reminding medicines intake, monitoring health values and symptoms. It allows sharing monthly reports and graphs with family and doctors.	Android, iOS	Yes	Yes	No	No	4	4
**IT15**	Farmavviso	https://play.google.com/store/apps/details?id=it.seekout.apps.farmadietto&hl=it	Care recipients	HEALTH	Not disease-specific	Therapy reminder app that allows users to set vocal or textual notifications to remember medication to be taken. It also acts as virtual 'medicine cabinet', keeping track of all the medicines stored at home.	Android, iOS	-	Yes	No	Yes	4	3
**IT17**	Lesioni da decubito - Lite	https://itunes.apple.com/it/app/lesioni-da-decubito-lite/id1093887592?mt=8	Carers	HEALTH	Not disease-specific	This app provides basic wound care notions based on the latest guidelines and available scientific evidence on pressure sores management. The app is designed and created by Dr. Marco Romitelli.	Android, iOS	Yes	-	No	Yes	4	3

Country	Name	Link	Target	Category	Disease/ condition	Description	Op. system	Reliability	Privacy	Registration	Connection	MARS	Score
**IT18**	DrDrin	https://play.google.com/store/apps/details?id=com.biocare.drdrin	Carers & care recipients	HEALTH	Not disease-specific	This app includes drugs reminder and planning; moreover, it gives the possibility to insert the amount of medicines bought and receive a notification when they are ending. It allows communication with some "tutor", like family members and doctors.	Android, iOS	Yes	Yes	Yes	Yes	4	4
**IT19**	Banca Dati Farmaci AIFA	https://itunes.apple.com/it/app/banca-dati-farmaci/id871252691?mt=8	Care recipients	HEALTH	Not disease-specific	Official drug database of the Italian Medicines Agency (AIFA). It allows downloading information sheets for all the pharmacological products authorized for sale in Italy.	Android, iOS	Yes	Yes	Yes	Yes	3	3
**IT20**	Automedicazione	https://play.google.com/store/apps/details?id=automedicazione.goticoweb&hl=it	General public	HEALTH	Not disease-specific	This app provides information about symptoms, disorders, drugs and active principles, allowing users to manage at best OTC drugs.	Android, iOS	Yes	-	Yes	No	4	3
**IT21**	Io equivalgo	https://play.google.com/store/apps/details?id=it.farmadati.cafarm&hl=it	General public	HEALTH	Not disease-specific	This app helps find equivalent medications searching by product name, active ingredients or using the barcode of the medicine box. The list of low-cost equivalent drugs is provided by the Italian Medicines Agency (AIFA).	Android, iOS	Yes	-	Yes	Yes	4	3

Country	Name	Link	Target	Category	Disease/ condition	Description	Op. system	Reliability	Privacy	Registration	Connection	MARS	Score
IT22	Bere acqua promemoria	https://play.google.com/store/apps/details?id=com.northpark.drinkwater&hl=it&rdid=com.northpark.drinkwater	General public	HEALTH	Not disease-specific	This app calculates how much water you should drink per day based on your weight and sets daily goals accordingly. It allows sending drinking reminders and monitoring body weight.	Android	-	-	Yes	No	4	4
IT23	Your Water - free	https://itunes.apple.com/ua/app/yourwater-free-your-water/id990678187?mt=8	General public	HEALTH	Not disease-specific	This app allows taking under control water intake by setting daily goals of water consumption, sending notifications to remind drinking, and tracking the progress towards the target.	iOS	-	-	Yes	Yes	4	3
IT24	Asma	https://play.google.com/store/apps/details?id=it.euromultimedia.asma&feature=search_result#?t=W251bGwsMSwyLDEsIml0LmV1cm9tdWx0aW1lZGlhLmFzbWEiXQ	General public	HEALTH	Respiratory	This App gives information on asthma and allows users to assess periodically their disease status. It provides a daily reminder for the drug therapy and information on the pollen concentration.	Android, iOS	Yes	Yes	No	No	3	3
IT25	Neglect App	https://itunes.apple.com/it/app/neglect-app/id788480837?mt=8	Care recipients	HEALTH	Stroke	This App uses virtual reality to rehabilitate neglect, one of the stroke's possible consequences.	iOS	-	Yes	No	No	4	4

Country	Name	Link	Target	Category	Disease/ condition	Description	Op. system	Reliability	Privacy	Registration	Connection	MARS	Score
IT26	Memorado	https://play.google.com/store/apps/details?id=com.memorado.brain.games	General public	HEALTH	Stroke, Neurodegenerative	This App contains memory games and other activities designed to train different cognitive functions such as attention and processing speed of information.	Android, iOS	Yes	Yes	No	No	3	4
IT27	Neuro Nation	https://play.google.com/store/apps/details?id=air.nn.mobile.app.main	General public	HEALTH	Stroke, Neurodegenerative	This App contains memory games and other activities designed to train different cognitive functions such as attention and processing speed of information.	Android, iOS	Yes	Yes	No	No	3	3
IT28	Alzheimer App	https://play.google.com/store/apps/details?id=com.alzheimer https://itunes.apple.com/us/app/alzheimer-app/id539167137?mt=8	Carers & care recipients	HEALTH	Neurodegenerative	This App is devoted to people with Alzheimer's disease, their relatives and caregiver. It gives a lot of information on Alzheimer's disease and advice on how to take care of people with this problem.	Android, iOS	-	Yes	No	No	4	4
IT29	Family GPS locator My family	https://play.google.com/store/apps/details?id=net.prtm.myfamily&hl=it	General public	TECHNOLOGIES	Neurodegenerative, Mental	This app allows locating family members and friends using GPS. It sends a notification when a person gets to or leave selected places and it has a group chat.	Android, iOS	-	Yes	Yes	Yes	3	3
IT30	Trova e proteggi i familiari (FamiloNet su iTunes)	https://play.google.com/store/apps/details?id=net.familo.android&rdid=net.familo.android	General public	TECHNOLOGIES	Neurodegenerative, Mental	This app allows locating family members and friends using GPS. It includes group chats with the possibility to create photo album. Moreover, it has a panic button to send an immediate alarm to all family members in case of danger.	Android, iOS	-	-	Yes	Yes	4	3

Country	Name	Link	Target	Category	Disease/ condition	Description	Op. system	Reliability	Privacy	Registration	Connection	MARS	Score
**IT31**	iNonni	https://play.google.com/store/apps/details?id=it.fab_lab.app.inonni&rdid=it.fab_lab.app.inonni	General public	TECHNOLOGIES	Not disease-specific	This app is addressed to all older adults and contains games, news, some learning activities, and information about pharmacies and common drugs. Moreover, there is the possibility to use this app for communicate using messages, calls and video calls using a simple design interface.	Android, iOS	-	Yes	No	Yes	4	4
**IT32**	Pharmawizard - Farmaci - Farmacie	https://play.google.com/store/apps/details?id=it.datawizard.pharmawizard.app	General public	CONTACTS	Not disease-specific	This app gives information on different drugs, compares the prices, and shows the equivalent ones. It also helps to find opened pharmacies.	Android, iOS	-	-	Yes	Yes	4	4
**IT33**	Salva una vita	https://play.google.com/store/apps/details?id=com.gag.salvaunavita&hl=it https://itunes.apple.com/it/app/salva-una-vita/id1020170957?mt=8	General public	HEALTH	Not disease-specific	This App provides useful information on facing medical emergencies.	Android, iOS	Yes	Yes	No	No	4	5

Country	Name	Link	Target	Category	Disease/ condition	Description	Op. system	Reliability	Privacy	Registration	Connection	MARS	Score
**IT34**	No barriere	https://www.associazionelucacoscioni.it/notizie/comunicati/app-no-barriere/	General public	TECHNOLOGIES	Not disease-specific	This app aims to enhance the mobility of persons with disabilities by using a geolocation system who collects reports about architectural and sensorial barriers. Each user can contribute to mapping the barriers adding notifications in the database and sending notice to local authorities.	Android, iOS	-	Yes	No	Yes	4	4
**IT35**	LIS	https://play.google.com/store/apps/details?id=appinventor.ai_sanny4_SB.LIS&hl=it	Carers & care recipients	TECHNOLOGIES	Sense	This App includes more than 150 words translated into sign language. It contains some videos that help the user to repeat the sign correctly.	Android	Yes	Yes	No	Yes	3	4
**IT36**	Spreadthe sign - il più grande dizionario della lingua dei segni	https://play.google.com/store/apps/details?id=com.spreadthesign.androidapp_paid	Carers & care recipients	TECHNOLOGIES	Sense	"Spread the sign" is a sign language dictionary. It contains many words in 20 different languages.	Android, iOS	Yes	Yes	No	Yes	3	4
**IT37**	La mia voce	https://play.google.com/store/apps/details?id=com.Zadig.LaMiaVoce	Carers & care recipients	TECHNOLOGIES	Stroke, Cancer, Sense	This App is a communication tool designed for people impaired to speak (for example in case of aphasia, vocal cords problems or speech difficulties). It helps people unable to speak to express their desires, requests, opinions and emotions. Several functions let the user customize the app to respond more and more to the different daily needs.	Android, iOS	Yes	Yes	Yes	No	4	3

Country	Name	Link	Target	Category	Disease/ condition	Description	Op. system	Reliability	Privacy	Registration	Connection	MARS	Score
**IT38**	Training Autogeno	https://itunes.apple.com/it/app/training-autogeno/id464008177?mt=8	General public	WELL-BEING	Not disease-specific	This App gives basic information on what the autogenous training is, and presents different exercises.	iOS	Yes	Yes	No	No	3	3
**IT39**	Esercizi di Respirazione Profonda Tecniche Pranayama	https://itunes.apple.com/it/app/esercizi-respirazione-profonda-tecniche-pranayama/id1135818237?mt=8	General public	WELL-BEING	Not disease-specific	This App presents different breathing exercises to promote relaxation.	iOS	-	Yes	Yes	No	3	3
**IT40**	WellBeing	https://itunes.apple.com/it/app/wellbeing/id586165211?mt=8	General public	WELL-BEING	Not disease-specific	This App presents guided meditations to promote relaxation and help users falling asleep.	iOS	-	Yes	No	No	3	3
**IT41**	Spiritual Me, meditazione, tecniche di consapevolezza, alleviamento dello stress	https://play.google.com/store/apps/details?id=com.spiritualmeapp.masters&hl=it	General public	WELL-BEING	Not disease-specific	This App presents guided meditations in order to promote relaxation and awareness.	Android, iOS	-	Yes	No	No	3	3

Country	Name	Link	Target	Category	Disease/ condition	Description	Op. system	Reliability	Privacy	Registration	Connection	MARS	Score
IT42	Training Autogeno APP	https://play.google.com/store/apps/details?id=appinventor.ai_kirpykirpy.TRAINING_AUTOGENO_APP	General public	WELL-BEING	Not disease-specific	This App gives basic information on what autogenous training is and presents different exercises.	Android	-	-	No	No	3	3
CY 01	Zoom Cyprus	https://play.google.com/store/apps/details?id=com.trackandtalk.android&hl=el	General public	CONTACTS	Not disease-specific	Whether you are living in Cyprus or just visiting, Zoom Cyprus has you covered. Offline GPS navigation with maps covering the entire island, up-to-date point-of-interest listings, as well as Emergency Services, in cooperation with the biggest insurance providers in Cyprus, that will give you the peace of mind you need to make the most of your experience.	Android	-	-	No	Yes	3	3
CY 02	Cyprus Pharmacies	https://play.google.com/store/apps/details?id=com.appbaker.app284162&hl=el	General public	CONTACTS	Not disease-specific	A simple app providing information about the pharmacies in Cyprus	Android, iOS	-	-	No	Yes	3	3
CY 03	Cyprus Pharmacies guide	https://play.google.com/store/apps/details?id=com.aspectsense.pharmacyguidecy	General public	CONTACTS	Not disease-specific	A simple app providing information about the pharmacies in Cyprus	Android, iOS	-	-	No	Yes	3	3
CY 04	map cyprus	http://www.greekapps.info/2013/01/map-cyprus.html	General public	CONTACTS	Not disease-specific	This is a useful touristic guide, including all information about Cyprus: pharmacies, hospital, taxies, gas stations etc. This app could be of	Android	Yes	-	Yes	Yes	4	4

Country	Name	Link	Target	Category	Disease/ condition	Description	Op. system	Reliability	Privacy	Registration	Connection	MARS	Score
						use for carers looking for hospitals and pharmacies.							
CY 05	Talking point forum	https://play.google.com/store/apps/details?id=com.tapatalk.forumalzheimersorguk&hl=el	Carers	CONTACTS	Neurodegenerative	Talking point forum has been developed by Alzheimer's Society UK. The users' needs to create an account to be able to post in the forum and create a network with other carers to communicate. Carers are able to read others' experiences, ask for advice, share information, join in discussions and, most of all, feel supported.	Android, iOS	Yes	-	Yes	Yes	3	4
CY 06	iCare health monitor	https://play.google.com/store/apps/details?id=comm.cchong.BloodAssistant&hl=el	Care recipients	HEALTH	Cardiovascular, Sense	This application is a useful tool to measure easily vision, hearing, blood pressure, oxygen, heart rate. The Greek translation is automatic; so many words are not well translated. The application has the technology to calibrate the measurements according to the data the users enter	Android, iOS	-	Yes	Yes	No	5	5
CY 07	Medication reminder	http://www.greekapps.info/2016/01/blog-post_20.html	General public	HEALTH	Not disease-specific	This is a very simple app, to remind medication. The language is Greek and is available only for Android system	Android	-	-	Yes	No	3	3
CY 08	Wheelmap	http://myhealthapps.net/app/details/124/Wheelmap	Care recipients	TECHNOLOGIES	Not disease-specific	The application shows map and all accessible places. Users can also upload photos and add comments to places	Android, iOS	Yes	-	Yes	Yes	4	4
CY 09	BIG Launcher (Free version)	http://myhealthapps.net/app/details/131/BIG-Launcher	General public	TECHNOLOGIES	Sense	A useful app to simplify the mobile touchscreen. The application has settings to enlarge the interface and the images on the screen. Suitable	Android	Yes	Yes	No	No	4	4

Country	Name	Link	Target	Category	Disease/ condition	Description	Op. system	Reliability	Privacy	Registration	Connection	MARS	Score
						android interface for older people, kids, people with visual impairments. More features are available with purchase of different apps							
**CY 10**	Dianoia	https://play.google.com/store/apps/details?id=org.scify.dianoia.app&hl=el	Carers & care recipients	HEALTH	Neurodegenerative	An application for people with mild cognitive impairment or mild dementia. Together with the carer, the care recipient can start suggested activities and cognitive stimulation activities to improve mode and cognitive abilities.	Android	Yes	No	No	No	4	4
**GR 01**	XrySOS	https://itunes.apple.com/gr/app/xrysos-pharmakeia-nosokomeia/id525751345?l=el&mt=8	General public	CONTACTS	Not disease-specific	This application provides all pharmacies and Hospitals in distance available in the specific date of search.	Android, iOS	Yes	No	Yes	Yes	3	3
**GR 02**	SOS calls	http://www.greekapps.info/2013/06/blog-post_7131.html	General public	CONTACTS	Not disease-specific	Users of this application may call an emergency number in case of accident, fire or any other emergency event. Do not use the application without purpose. In case you call 112 by mistake, do not hang the phone, instead wait to confirm that this was not intentionally.	Android	Yes	-	Yes	No	4	4
**GR 03**	SOS phones	http://www.greekapps.info/2013/10/blog-post_6922.html	General public	CONTACTS	Not disease-specific	This application helps you to have access to useful SOS phones including emergency numbers and websites	Android	Yes	-	Yes	No	4	3

Country	Name	Link	Target	Category	Disease/ condition	Description	Op. system	Reliability	Privacy	Registration	Connection	MARS	Score
**GR 04**	Farmakeia	https://appapp.io/us/app/farmakeia/364925463	General public	CONTACTS	Not disease-specific	This application provides all pharmacies and Hospitals in Greece available in near distance taking the GPS signal of the current location.	iOS	Yes	-	Yes	Yes	4	4
**GR 05**	ifarmakeia	http://www.greekapps.info/2016/09/ifarmakeia.html	General public	CONTACTS	Not disease-specific	An application for finding the nearest pharmacy. It provides details on products sales.	Android	-	-	Yes	Yes	4	3
**GR 06**	Talking point forum	https://play.google.com/store/apps/details?id=com.tapatalk.forumalzheimersorguk&hl=el	Carers	CONTACTS	Neurodegenerative	Talking point forum has been developed by Alzheimer's Society UK. The users' needs to create an account to be able to post in the forum and create a network with other carers to communicate. Carers are able to read others' experiences, ask for advice, share information, join in discussions and, most of all, feel supported.	Android, iOS	Yes	-	Yes	Yes	3	4
**GR 07**	iCare health monitor	https://play.google.com/store/apps/details?id=comm.cchong.BloodAssistant&hl=el	Care recipients	HEALTH	Cardiovascular, Sense	This application is a useful tool to measure easily vision, hearing, blood pressure, oxygen, heart rate. The Greek translation is automatic; so many words are not well translated. The application has the technology to calibrate the measurements according to the data the users enter	Android, iOS	-	Yes	Yes	No	5	5
**GR 08**	Medication reminder	http://www.greekapps.info/2016/01/blog-post_20.html	General public	HEALTH	Not disease-specific	This is a very simple app, to remind medication. The language is Greek and is available only for Android system	Android	-	-	Yes	No	3	3
**GR 09**	Wheelmap	http://myhealthapps.net/app/details/124/Wheelmap	Care recipients	TECHNOLOGIES	Not disease-specific	The application shows map and all accessible places. Users can also	Android, iOS	Yes	-	Yes	Yes	4	4

Country	Name	Link	Target	Category	Disease/ condition	Description	Op. system	Reliability	Privacy	Registration	Connection	MARS	Score
						upload photos and add comments to places							
GR 10	BIG Launcher (Free version)	http://myhealthapps.net/app/details/131/BIG-Launcher	General public	TECHNOLOGIES	Sense	A useful app to simplify the mobile touchscreen. The application has settings to enlarge the interface and the images on the screen. Suitable android interface for older people, kids, people with visual impairments. More features are available with purchase of different apps	Android	Yes	Yes	No	No	4	4
GR 11	Dianoia	https://play.google.com/store/apps/details?id=org.scify.dianoia.app&hl=el	Carers & care recipients	HEALTH	Neurodegenerative	An application for people with mild cognitive impairment or mild dementia. Together with the carer, the care recipient can start suggested activities and cognitive stimulation activities to improve mode and cognitive abilities.	Android	Yes	No	No	No	4	4
CY GR 01	Alz & Dementia, Alzheimer's Daily companion	https://play.google.com/store/apps/details?id=com.homeinstead.alzheimersassistantandroid&hl=el	Carers	HEALTH	Neurodegenerative	ENGLISH RESOURCE<br>This app provides immediate tips and practical advice for all of the behaviours and situations carers face on a daily basis. (500 searchable tips and practical solutions) Caregivers can search on situations they are struggling. The tips are compiled by experts as well as family caregivers dealing with Alzheimer's or other dementia illnesses. Features Include:<br>• advice and tips rated by carers<br>• 24-hour caregiving assistance available via toll free phone number or email submission.	Android, iOS	Yes	No	No	No	4	4

Country	Name	Link	Target	Category	Disease/ condition	Description	Op. system	Reliability	Privacy	Registration	Connection	MARS	Score
						• Access to free Alzheimer's and other dementias caregiver resources and training materials.<br>• Caregivers sharing their own advice.							
CY GR 02	Dementia Support	https://play.google.com/store/apps/details?id=io.appery.project93137&hl=el	Carers	HEALTH	Neurodegenerative	ENGLISH RESOURCE<br>This application aims to offer information and advice to carers of people with dementia to increase the quality of life. The app will offer insight on the challenges and difficulties that face those affected by dementia and those around them. The users have to select from a menu the topic of interest: support, inspiration, dementia, and network.	Android, iOS	Yes	No	Yes	Yes	4	4
CY GR 03	S3 Care giver wellness	https://play.google.com/store/apps/details?id=com.nexle.s3caregiver&hl=el	Carers	WELL-BEING	Stroke	ENGLISH RESOURCE<br>This is an application developed for the S3 (Stroke Support Station) by Dr. Melvyn Zhang, Dr. Roger Ho & Dr Leonard Yeo. The app provides information on stroke, relevant videos about mindfulness-based therapy, S3 Podcast about Care-giving and Carers' Well-being and enables carers to keep track of their mental health well-being using validated questionnaires.	Android	Yes	-	Yes	No	4	4
CY GR 04	Parkinson's EasyCall	https://play.google.com/store/apps/details?id=co.uk.org.parkinsons&hl=el	General public	TECHNOLOGIES	Not disease-specific	ENGLISH RESOURCE<br>This application is a speed dial app, to call carers and friends easily without searching in the phone directory. Numbers can be entered manually or added directly from your contacts list.	Android	Yes	-	Yes	No	3	3

Country	Name	Link	Target	Category	Disease/ condition	Description	Op. system	Reliability	Privacy	Registration	Connection	MARS	Score
						Contact buttons are created as and when needed as speed dials start to fill up. For people who have dexterity issues and those who do not, the Easy Call app will make calling your important contacts much simpler, easier and a hassle-free process.							
CY GR 05	Asthma Australia	https://itunes.apple.com/au/app/asthma-australia-asthma-app/id1057041779?mt=8	Carers & care recipients	HEALTH	Respiratory	ENGLISH RESOURCE<br>An educational tool developed by Asthma Australia for healthcare professionals and people with asthma (and their families and carers). It provides easy access to information and resources about asthma, asthma medications, device technique videos, asthma action plans and asthma first aid and clinical guidelines.	iOS	Yes	-	Yes	No	4	4
CY GR 06	Cancer.Net Mobile	https://itunes.apple.com/us/app/cancer.net-mobile/id433501257?mt=8	Carers & care recipients	HEALTH	Cancer	ENGLISH RESOURCE<br>An application for monitoring your cancer by finding up to date information, record questions, log medications, track symptoms, scheduling, link questions	iOS	Yes	Yes	Yes	No	4	5
CY GR 07	Canceraid	https://play.google.com/store/apps/details?id=au.com.canceraid	Carers & care recipients	HEALTH	Cancer	ENGLISH RESOURCE<br>CancerAid is a first-of-its kind tool that helps anyone diagnosed with cancer navigate through each stage of his/her cancer journey. The app brings patients and caregivers greater structure in their lives by providing them with an easy to use organisational platform and a medically reliable source of cancer information.	Android, iOS	-	Yes	No	Yes	4	3

Country	Name	Link	Target	Category	Disease/ condition	Description	Op. system	Reliability	Privacy	Registration	Connection	MARS	Score
**CY GR 08**	Care4Dementia	https://play.google.com/store/apps/details?id=au.edu.unsw.Care4Dementia&hl=el	Carers	HEALTH	Neurodegenerative	ENGLISH RESOURCE<br>This application is based on the document Behaviour Management-A Guide to Good Practice: Managing Behavioural and Psychological Symptoms of Dementia (2012) by Collaborative Research Centre (DCRC-ABC). The app provides information and advice to carers in their role of caring for persons with behavioural changes that can occur in dementia. Information on what these behaviours look like, why they might be happening and what you can do to help is included for those most commonly reported by carers. Understanding why behavioural changes are occurring can help to manage the behaviour.	Android, iOS	Yes	No	Yes	No	4	4
**CY GR 09**	constipation learning	https://itunes.apple.com/us/app/constipation-learning/id1069767371?mt=8	Care recipients	HEALTH	Digestive	ENGLISH RESOURCE<br>This application will inform patients and providers about 1) normal process of defecation and 2) neuromuscular coordination & biofeedback training exercises to learn the normal process of defecation	iOS	Yes	-	Yes	No	3	3
**CY GR 10**	DementiAssist	https://play.google.com/store/apps/details?id=com.bhcsclip.dementiassist.activity&hl=el	Carers	HEALTH	Neurodegenerative	ENGLISH RESOURCE<br>The application supports carers on how to approach a PWD, behavioural disorders are discussed (aggression and Anger agitation or anxiety, apathy, confusion, pacing, problematic	Android, iOS	Yes	-	Yes	No	4	5

Country	Name	Link	Target	Category	Disease/ condition	Description	Op. system	Reliability	Privacy	Registration	Connection	MARS	Score
						vocalization, psychosis, resistance to bathing, resistance to dressing, resistance to feeding, resistance to oral carer, rummaging or stealing, sexually inappropriate behaviour, sleeping problems, sundowning and wandering. All these behaviours are presented through 4 trigger areas: physical, emotional, social, and environmental. When the user selects the most relevant trigger, actions are presented to manage the symptoms							
CY GR 11	heart health - controlling high blood pressure and cholesterol to reduce cardiac risk factors	https://itunes.apple.com/us/app/heart-health-controlling-high/id806004909?mt=8	General public	HEALTH	Cardiovascular	ENGLISH RESOURCE<br>An application proving general information on heart attack, heart diseases. Learn all about:<br>– Cholesterol and how to manage it<br>– Healthy dietary decisions<br>– Prevention of heart attacks<br>– Living with a heart condition<br>– Signs and symptoms of common ailments<br>– Reducing your risk<br>– How to handle high blood pressure<br>Self-monitoring your blood pressure.	iOS	Yes	-	Yes	No	3	3
CY GR 12	House of memories	https://itunes.apple.com/gb/app/my-house-of-memories/id847763460?mt=8	Carers & care recipients	HEALTH	Neurodegenerative	ENGLISH RESOURCE<br>House of memories is an application developed for people with memory disorders and their carers. The app is a reminiscence-training programme, designed to support people living with dementia and their carers. The programme is based on the fantastic	iOS	Yes	-	Yes	Yes	4	4

Country	Name	Link	Target	Category	Disease/ condition	Description	Op. system	Reliability	Privacy	Registration	Connection	MARS	Score
						objects, archives and stories at the Museum of Liverpool.							
**CY GR 13**	iStand Falls prevention	https://itunes.apple.com/us/app/istand-falls-prevention-exercise/id587885952?mt=8	Carers & care recipients	HEALTH	Musculoskeletal	ENGLISH RESOURCE<br>This application provides information and exercises on fall prevention. Helpful videos facilitate the exercises	iOS	Yes	-	Yes	No	3	4
**CY GR 14**	Living and Dying well with Dementia	https://itunes.apple.com/kw/app/living-dying-well-dementia/id945365520?mt=8	Carers	HEALTH	Neurodegenerative	ENGLISH RESOURCE<br>The application introduces key issues in dementia and end of life care. Interestingly uses the example of Jill to present possible behaviours, reaction of the person with dementia and possible solutions using tips, exercises and resources. The app has been developed by a partnership including Alzheimer's Society UK, university of Chester, Frontline communication and other associations	iOS	Yes	-	Yes	No	4	3
**CY GR 15**	Memory box	https://play.google.com/store/apps/details?id=com.sci.memorybox&hl=el	Carers & care recipients	HEALTH	Neurodegenerative	ENGLISH RESOURCE<br>Memory box is a smartphone application that aims to serve as a memory aid and a conversation inspiration to support relatives and caregivers of those suffering from Alzheimer’s or other types of dementia. It contains visual, musical and written tips for conversations and memory support. The app contains information pieces about famous events, people, places and topics from the 20th century and the user can save your favourite memories in a scrapbook.	Android, iOS	Yes	Yes	No	No	5	5

Country	Name	Link	Target	Category	Disease/ condition	Description	Op. system	Reliability	Privacy	Registration	Connection	MARS	Score
CY GR 16	Mood tools	https://play.google.com/store/apps/details?id=com.moodtools.moodtools&hl=el	General public	HEALTH	Mental	ENGLISH RESOURCE MoodTools contains Thought Diary - Activities - based on Behavioural Activation Therapy. Safety Plan - Information - Test – and videos. MoodTools was designed in collaboration with multiple mental health professionals.	Android, iOS	Yes	No	Yes	No	4	4
CY GR 17	Multiple sclerosis	https://play.google.com/store/apps/details?id=com.sunmediasoft.sclerosis&hl=el	Carers & care recipients	HEALTH	Neurodegenerative	ENGLISH RESOURCE The application provides basic info on Multiple Sclerosis, such as a collection of articles on the definition, the symptoms, prevalence, diagnosis, carers, treatments, causes, chronic pain, and advice.	Android	-	No	Yes	No	3	3
CY GR 18	Pacifica	https://itunes.apple.com/us/app/pacifica-anxiety-stress-depression-relief/id922968861?mt=8	General public	WELL-BEING	Not disease-specific	ENGLISH RESOURCE Pacifica gives you psychologist-designed tools to address them based on Cognitive Behavioural Therapy, mindfulness meditation, relaxation, and mood/health tracking.	Android, iOS	-	Yes	No	No	4	3
CY GR 19	Relaxation techniques	https://play.google.com/store/apps/details?id=com.psiholosko_savetovaliste.relaksacija&hl=en	General public	WELL-BEING	Not disease-specific	ENGLISH RESOURCE An application to facilitate progressive muscle relaxation.	Android	Yes	-	No	No	4	3
CY GR 20	Self-help anxiety management	https://play.google.com/store/apps/details?id=com.uwe.myoxygen&hl=el	General public	WELL-BEING	Mental	ENGLISH RESOURCE The app Self-help anxiety management is a way to monitor anxiety using graphical display, 25 self-help options covering: information about anxiety, thinking and anxiety, physical relaxation, mental relaxation, health	Android, iOS	Yes	No	No	No	4	4

Country	Name	Link	Target	Category	Disease/ condition	Description	Op. system	Reliability	Privacy	Registration	Connection	MARS	Score
						and anxiety, guidance on putting self-help into practice, self-monitoring of anxiety, closed social network of same users, external links							
**PT 01**	Knok - Médicos ao domicílio	https://itunes.apple.com/pt/app/knok-medicos-ao-domicilio/id1060604041?mt=8	General public	CONTACTS	Not disease-specific	Allows calling a doctor home.	iOS	Yes	-	No	Yes	4	5
**PT 02**	My Medi Stuff	https://itunes.apple.com/pt/app/my-medi-stuff/id596482153?mt=8	General public	CONTACTS	Not disease-specific	Allows managing medical appointments and medical contacts, both from doctors and patients.	iOS	-	-	No	No	4	4
**PT 03**	Emergência App 112	https://play.google.com/store/apps/details?id=pt.applyab.app112&hl=pt_PT	Care recipients	CONTACTS	Not disease-specific	Emergency app 112 is an application specially developed for the inclusion of people with disabilities, deaf and elderly people, is intended to facilitate the communication of users with personal assistance services in cases of medical emergencies, personal accidents, car breakdown, earthquake, Terrorist, bullying, domestic violence, etc.	Android	Yes	-	Yes	Yes	4	4
**PT 04**	Farmácias de Serviço (On-duty Pharmacies)	https://play.google.com/store/apps/details?id=com.milempresas.farmacias&hl=pt_PT	General public	CONTACTS	Not disease-specific	Pharmacy locator	Android, iOS	-	-	Yes	No	4	4
**PT 05**	Cancro Colorrectal	https://itunes.apple.com/pt/app/cancro-	General public	HEALTH	Cancer	Application on Colorectal Cancer Prevention. The app presents information on Colorectal Cancer,	Android, iOS	Yes	-	Yes	No	4	4

Country	Name	Link	Target	Category	Disease/ condition	Description	Op. system	Reliability	Privacy	Registration	Connection	MARS	Score
		colorrectal/id1159657302?l=en&mt=8				focusing on aspects related to epidemiology, prevention, treatment and support.							
**PT 06**	Cancro Mama	https://itunes.apple.com/pt/app/cancro-mama/id1025046154?l=en&mt=8	General public	HEALTH	Cancer	Application on Breast Cancer Prevention. The app presents information on Breast Cancer, focusing on aspects related to epidemiology, prevention, treatment and support for women with breast cancer.	Android, iOS	Yes	-	Yes	No	4	4
**PT 07**	Elderly Care	http://elderlycareapp.com	Carers	HEALTH	Cardiovascular, Stroke, Neurodegenerative, Cancer, Musculoskeletal, Diabetes, Mental, Sense, Incontinence	It contains information on how to relate to your loved ones and how to cope with the different impairments that may occur as you age. It aims to offer you both inspiration and information on how you and your relative can make life a little easier and more enjoyable.	Android, iOS	Yes	-	Yes	Yes	4	4
**PT 08**	Diabetes: M	https://play.google.com/store/apps/details?id=com.mydiabetes&hl=pt_PT	Care recipients	HEALTH	Diabetes	This application is intended to help diabetics to manage better their diabetes and keep it under control. Users can log their values in this diary and keep the records with them all the time. The application tracks almost all aspects of the diabetes treatment and provides detailed reports, charts and	Android	Yes	Yes	Yes	No	4	4

Country	Name	Link	Target	Category	Disease/ condition	Description	Op. system	Reliability	Privacy	Registration	Connection	MARS	Score
						statistics to share via the email with the supervising physician. It provides various tools to the diabetics, so they can find the trends in blood glucose levels and allows users to calculate normal and prolonged insulin boluses using its highly effective, top-notch bolus calculator.							
**PT 09**	Senior Mood Assessment - Depressed or Not?	https://itunes.apple.com/pt/app/senior-mood-assessment-depressed/id430169679?mt=8	Care recipients	HEALTH	Mental	ENGLISH RESOURCE<br>Questionnaire to evaluate depression.	iOS	-	-	Yes	No	3	3
**PT 10**	Dementia Support	https://play.google.com/store/apps/details?id=io.appery.project93137	Carers	HEALTH	Neurodegenerative	ENGLISH RESOURCE<br>The application contains information and support, as well as specific advice and tips for those wishing to learn more about dementia; the practical approach and the challenges people face when caring for someone in a home environment.<br>The application is aimed at family and friends of dementia patients and aims to offer support for those caring for family members in the home.	Android, iOS	Yes	-	Yes	Yes	4	4
**PT 11**	Keys to Care	https://itunes.apple.com/gb/app/keys-to-care/id935587341?mt=8	Carers	HEALTH	Neurodegenerative, Incontinence	ENGLISH RESOURCE<br>It gives inspirational, practical guidance to help improve the health and wellbeing of those one looks after.		Yes	-	Yes	No	3	3

Country	Name	Link	Target	Category	Disease/ condition	Description	Op. system	Reliability	Privacy	Registration	Connection	MARS	Score
**PT 12**	Care4Dementia	https://play.google.com/store/apps/details?id=au.edu.unsw.Care4Dementia	Carers	HEALTH	Neurodegenerative, Mental	ENGLISH RESOURCE<br>This App was developed to provide information and support for carers in their role of caring for persons with behavioural changes that can occur in dementia. Information on what these behaviours look like, why they might be happening and what you can do to help is included for those most commonly reported by carers. Understanding why behavioural changes are occurring can help to manage the behaviour. By downloading this App for carers, you agree to the disclaimer below.	Android, iOS	Yes	-	Yes	No	4	4
**PT 13**	Carezone	https://itunes.apple.com/pt/app/carezone-health-info-organizer/id829841726?mt=8	Carers	HEALTH	Not disease-specific	ENGLISH RESOURCE<br>CareZone makes it simpler to take care of yourself, an aging parent, and other family members that need your help. It allows to manage medication and to track pains, sleep, medication, mood.	Android, iOS	Yes	Yes	No	Yes	4	4
**PT 14**	Symptomate	https://play.google.com/store/apps/details?id=com.symptomate.mobile	General public	HEALTH	Not disease-specific	ENGLISH RESOURCE<br>Symptom checker designed by doctors that will help to find out what may be going on with your health.	Android, iOS	Yes	-	No	No	4	4
**PT 15**	Tactio	https://itunes.apple.com/us/app/tactio-health-my-connected/id477774973?mt=8	General public	HEALTH	Not disease-specific	Tactio Health App helps to track and manage a wide range of health data by manual logging or syncing with connected health apps and medical devices. It allows tracking weight, steps, nutrition, activity, sleep, mood,	Android, iOS	Yes	-	No	Yes	4	4

Country	Name	Link	Target	Category	Disease/ condition	Description	Op. system	Reliability	Privacy	Registration	Connection	MARS	Score
						blood pressure, pulse, glucose, cholesterol, temperature and oximetry.							
**PT 16**	Elderly Mobility Scale	https://itunes.apple.com/pt/app/elderly-mobility-scale/id722895882?mt=8	Carers	HEALTH	Not disease-specific	ENGLISH RESOURCE<br>Questionnaire to evaluate mobility of elder people.	iOS	-	-	No	No	4	3
**PT 17**	CareZapp	https://itunes.apple.com/pt/app/carezapp/id935603605?l=en&mt=8	Carers	HEALTH	Not disease-specific	ENGLISH RESOURCE<br>Enables caregivers to create a private network of care and help ensure communications and collaboration between all people involved in caring.	Android, iOS	-	-	No	Yes	3	3
**PT 18**	Senior Assist	https://itunes.apple.com/pt/app/senior-assist-free/id818337395?mt=8	General public	HEALTH	Not disease-specific	ENGLISH RESOURCE<br>A medication alarm to remind users to take their medication at the correct time. Also has a one-touch HELP button.	Android, iOS	-	-	No	No	4	4
**PT 19**	Cardiógrafo (Cardiograph)	https://play.google.com/store/apps/details?id=com.macropinch.hydra.android&hl=pt_PT	General public	HEALTH	Not disease-specific	The Cardiograph app measures users' heart rate and enables them to save data for future reference. Cardiograph calculates the heart rate using the built-in smartphone sensors and devices (camera, ...). Moreover, this app also has a multi-tracking function, which enables logging data of multiple people with individual profiles.	Android, iOS	Yes	-	Yes	No	4	4
**PT 20**	iCare Monitor de saúde	https://play.google.com/store/apps/details?id=comm.c	General public	HEALTH	Not disease-specific	ICare Health Monitor can measure blood pressure, heart rate, vision, hearing, SpO2, respiratory rate.	Android, iOS	Yes	-	Yes	Yes	3	4

Country	Name	Link	Target	Category	Disease/ condition	Description	Op. system	Reliability	Privacy	Registration	Connection	MARS	Score
		chong.BloodAssistant&hl=pt_PT											
**PT 21**	MediSafe Pill Reminder & Medication Tracker	https://play.google.com/store/apps/details?id=com.medisafe.android.client&referrer=af_tranid%3D0vUYkpoFfwDY9GwsNWbFPw%26pid%3Dbadgebuttons%26c%3DmedisafeBadgeButtons	General public	HEALTH	Not disease-specific	Medication manager and pill reminder. Provides the tools, support and information people need to take their medications the way they are supposed to.	Android, iOS	Yes	-	Yes	No	4	5
**PT 22**	Elder 411	http://www.elder411.net	Carers	HEALTH	Not disease-specific	ENGLISH RESOURCE<br>Pieces of expert advice are organized into 11 eldercare topics providing solutions to caregiving problems.	iOS	-	-	Yes	No	3	3
**PT 23**	Senior Balance Quiz	https://itunes.apple.com/pt/app/senior-balance-quiz-afraid/id499146406?mt=8	Care recipients	HEALTH	Not disease-specific	ENGLISH RESOURCE<br>Questionnaire to evaluate balance problems.	iOS	-	-	Yes	No	3	3
**PT 24**	Desordem e Doenças Dicionário	https://play.google.com/store/apps/details?id=com.quatkhoi.diseases.dictionary&hl=pt_PT	General public	HEALTH	Not disease-specific	Diseases Dictionary is a free list of medical disorders and diseases that provides all the information about symptoms, disorder and treatment and many medical terminologies.	Android, iOS	-	-	Yes	No	4	4
**PT 25**	Hora do Medicamento (Pill Time)	https://play.google.com/store/apps/details?id=com.ho	General public	HEALTH	Not disease-specific	The application enables users to enter the name of the medicine, the interval between doses and time of the first dose. The additional features to pay	Android, iOS	-	-	Yes	No	4	3

Country	Name	Link	Target	Category	Disease/ condition	Description	Op. system	Reliability	Privacy	Registration	Connection	MARS	Score
		radomedicamento&hl=pt_PT				for also lets users remove the advertisements, add an unlimited number of meds and choose different notification sounds for each pill.							
**PT 26**	Socorrismo	https://itunes.apple.com/pt/app/socorrismo-cruz-vermelha/id947395441?mt=8	General public	HEALTH	Cardiovascular, Stroke, Respiratory, Musculoskeletal, Diabetes, Mental	Useful information on first aid and how to deal with the most common emergencies of everyday life, through videos, interactive quizzes and simple advice.	Android, iOS	Yes	-	Yes	No	4	4
**PT 27**	eMed.pt	https://play.google.com/store/apps/details?id=com.codepixel.infarmedAndroid&hl=pt_PT	General public	HEALTH	Not disease-specific	eMED.pt app provides information regarding medicines - price, cheaper equivalents and leaflets. It has an alarm system, which enables users to set alarms that remind them to take or give the prescribed medication, and a feature that provides information about the nearby pharmacies.	Android, iOS	Yes	-	No	Yes	4	4
**PT 28**	Farmácias Portuguesas	https://play.google.com/store/apps/details?id=pt.anf.farmaciasportuguesas&hl=pt_PT	General public	HEALTH	Not disease-specific	Way to find the nearest open pharmacy, buy or reserve directly in the application of the Portuguese Pharmacies and lift at the pharmacy or receive at home. Get access to all the products and medications available in existing pharmacies. Also provides information on health aspects. Receive alerts at the right time to take your medicines or track the evolution	Android, iOS	Yes	-	No	Yes	4	4

Country	Name	Link	Target	Category	Disease/ condition	Description	Op. system	Reliability	Privacy	Registration	Connection	MARS	Score
						of your health data and know if it is in the recommended values. Access relevant health information content and improve your well-being on a daily basis.							
**PT 29**	MySNS	https://play.google.com/store/apps/details?id=pt.min saude.spms.mysns	General public	HEALTH	Not disease-specific	MySNS is a tool that allows you to consult news from the National Health Service, consult health information, provide a list and map of health institutions (Hospitals, Primary Health Care and Pharmacies), Health information line 24, notifications such as heat alerts, etc.,	Android, iOS	Yes	-	No	Yes	3	2
**PT 30**	Elder 911	http://www.elder911.net	Carers	HEALTH	Not disease-specific	ENGLISH RESOURCE<br>Pieces of expert advice providing solutions to caregiving problems.	iOS	-	-	Yes	No	4	4
**PT 31**	mycare	https://play.google.com/store/apps/details?id=com.drawandcode.mycare	Carers	WELL-BEING	Not disease-specific	ENGLISH RESOURCE<br>MyCare provides information and support to allow carers to work towards their own recovery and well-being. It contains elements of education and interaction to help carers achieve goals, manage stress and look after themselves.	Android	Yes	-	Yes	No	3	3
**PT 32**	Tender Loving Eldercare	https://itunes.apple.com/us/app/tender-loving-eldercare/id1118172662?mt=8	Carers	WELL-BEING	Neurodegenerative	ENGLISH RESOURCE<br>Articles, expert tips, resources and interviews with other family caregivers; inspiring words and motivating ideas to help caregivers.	iOS	-	-	Yes	Yes	3	3
**PT 33**	S3 Care-giver	https://play.google.com/store/apps/	Carers	WELL-BEING	Stroke	ENGLISH RESOURCE	Android, iOS	Yes	-	Yes	Yes	4	4

Country	Name	Link	Target	Category	Disease/ condition	Description	Op. system	Reliability	Privacy	Registration	Connection	MARS	Score
		details?id=com.nexle.s3caregiver&hl=pt_BR				1. Provide caregivers of stroke patients with necessary care-giving information; 2. Provide links to relevant videos about mindfulness based therapy to aid in the mental health well-being of caregivers (Please note that the access to mindfulness based therapy would require subscription to S3 via http://strokesupportstation.org.sg); 3. S3 Podcast about Care-giving and Carers' Well-being; 4. Enabling Caregivers to keep track of their mental health well-being using validated questionnaires.							
SE 01	Nollvision – Anhörig	https://play.google.com/store/apps/details?id=com.sdc_nollvision_relative.myapp&hl=sv	Carers	HEALTH	Neurodegenerative	The app aims at providing information and advice on dementia. Different contents available to illustrate the characteristics of dementia, how to manage daily issues and how to support the patient.	Android, iOS	Yes	Yes	No	No	3	3
SE 02	SENIORG	https://play.google.com/store/apps/details?id=se.sict.seniorg&hl=sv	Carers	TECHNOLOGIES	Not disease-specific	The app constitutes a communication tool between family members of a person living in a nursing home and the nursing staff. It allows family members to send and receive messages and obtain images of the older person. It works for nursing homes managed by the same company.	Android, iOS	Yes	Yes	No	Yes	3	3

Country	Name	Link	Target	Category	Disease/ condition	Description	Op. system	Reliability	Privacy	Registration	Connection	MARS	Score
**SE 03**	Doro Connect & Care®	https://play.google.com/store/apps/details?id=com.doro.cnc.mobile&hl=sv	Carers & care recipients	TECHNOLOGIES	Not disease-specific	The app aims at enabling a care network around the older person. It enables chat and group chat, emergency calls from the older person, reminders and to-do lists, and GPS monitoring.	Android, iOS	Yes	Yes	No	Yes	4	4
**SE 04**	Landräddningen	https://play.google.com/store/apps/details?id=se.elicit.posifon_landraddningen&hl=sv	Carers & care recipients	CONTACTS	Not disease-specific	The app enables functions for emergency calls and emergency networks around the older person. Through GPS tracker, alarm system and information sharing with other volunteers, the older person can contact, be monitored and supported for emergency situations.	Android, iOS	Yes	Yes	No	Yes	3	4
**SE 05**	Elderly Care	https://play.google.com/store/apps/details?id=com.sci.elderlycare&hl=sv	Carers	HEALTH	Not disease-specific	The app contains information, advice and stories for carers of older people. It aims to improve knowledge around most common issues on older people and family carers themselves.	Android, iOS	Yes	Yes	Yes	Yes	4	3
**SE 06**	Kalmar Omsorg	https://play.google.com/store/apps/details?id=eu.appbolaget.kalmaromsorg&hl=sv	Carers	CONTACTS	Not disease-specific	The app provides information about the offer of residential care in the municipality of Kalmar. It shows the facilities, fees, activities and conditions.	Android, iOS	Yes	Yes	Yes	Yes	3	2
**SE 07**	Safe+Sound?	https://play.google.com/store/apps/details?id=se.nobiggig.safeandsound&hl=sv	Care recipients	TECHNOLOGIES	Not disease-specific	The app sends an alert via SMS to a family member if the older person does not deactivate it (meaning he/she has some problem). It includes also a GPS tracker for monitoring position.	Android, iOS	Yes	Yes	Yes	Yes	3	3

Country	Name	Link	Target	Category	Disease/ condition	Description	Op. system	Reliability	Privacy	Registration	Connection	MARS	Score
**SE 08**	Första hjälpen	https://play.google.com/store/apps/details?id=com.trygghansa.forstahjalpen	General public	CONTACTS	Not disease-specific	The app provides information about how to respond in a crisis. Important phone numbers are provided, etc.	Android, iOS	Yes	-	Yes	Yes	4	5
**SE 09**	Röda korset-första hjälpen	https://play.google.com/store/apps/details?id=com.cube.gdpc.swe	General public	CONTACTS	Not disease-specific	The app provides information about what to do in a crisis. Acute illness, accidents or similar incidents, how to prevent accidents, and also crises when you are abroad.	Android, iOS	Yes	-	Yes	Yes	4	4
**SE 10**	Fritt fram	https://play.google.com/store/apps/details?id=se.frittfram.frittfram	Care recipients	CONTACTS, TECHNOLOGIES	Not disease-specific	In the app, you can check if museums, restaurants, etc. have adapted environments for people with disabilities concerning toilets, ramps, parking etc. The developer is a private company.	Android, iOS	-	-	No	Yes	4	5
**SE 11**	Toahjälpen	https://play.google.com/store/apps/details?id=magotarm.se.toahjlpen	Care recipients	CONTACTS, TECHNOLOGIES	Not disease-specific	This app helps you find nearest toilet, informing if the toilet is free and if it is for persons with disabilities. However, it only works in Stockholm, Gothenburg and Visby. It is for persons with digestive illnesses or it could be urinary incontinence as well.	Android, iOS	Yes	-	Yes	Yes	3	2
**SE 12**	Cancervård	https://play.google.com/store/apps/details?id=se.rcc.RCCApp.Android	Care recipients	HEALTH	Cancer	Information about cancer diagnoses that have a standardized care course.	Android, iOS	Yes	-	Yes	Yes	3	3
**SE 13**	Triabetes	https://play.google.com/store/apps/details?id=com.dia	Care recipients	HEALTH	Diabetes	Triabetes is an app where the patient can take control over his/her diabetes, self-care. You can document how you eat, work out, and take medicines	Android, iOS	Yes	Yes	No	Yes	4	3

Country	Name	Link	Target	Category	Disease/ condition	Description	Op. system	Reliability	Privacy	Registration	Connection	MARS	Score
		betestoolssweden.mmmminisystem				over time. You can fill in measures of your blood sugar and follow over time related to what you eat, how you work out etc. You can set up goals for the different variables.							
**SE 14**	tät	https://play.google.com/store/apps/details?id=se.umu.its.tat	General public	HEALTH	Incontinence	Information about different exercises concerning urinary incontinence when you cough, jump or physical effort. For women.	Android, iOS	Yes	-	Yes	Yes	4	5
**SE 15**	Reumatiker	https://play.google.com/store/apps/details?id=se.frisq.reumapp	Carers & care recipients	HEALTH	Musculoskeletal	This app is for people with rheumatism. It aims to gather different kinds of functions and information valuable to enable better control over the person's own health. The app provides news, viewing of prescriptions (patient and carer), order medicines, read your health care journal, report health measures to doctor via system called PER.	Android, iOS	Yes	Yes	No	Yes	4	3
**SE 16**	Memory box	https://play.google.com/store/apps/details?id=com.sci.memorybox	Carers	HEALTH	Neurodegenerative	The app provides written, visual and musical conversation tips on 20th Century that enables better interaction with the person with dementia.	Android, iOS	Yes	Yes	No	Yes	4	5
**SE 17**	Kronans apotek	https://play.google.com/store/apps/details?id=se.kronansapotek.kronan	General public	HEALTH	Not disease-specific	Helps you keep track of your prescribed medicines and gives a reminder when you need to renew your prescriptions. You can also choose a power of attorney if needed.	Android, iOS	Yes	Yes	No	Yes	5	5

Country	Name	Link	Target	Category	Disease/ condition	Description	Op. system	Reliability	Privacy	Registration	Connection	MARS	Score
**SE 18**	Medisafe medicinpåminnelser	https://play.google.com/store/apps/details?id=com.medisafe.android.client	Care recipients	HEALTH	Not disease-specific	The app is for self-care. It provides reminders concerning medicines and doctors' appointments. You can make measurements of blood pressure, pulse, glucoses, pain etc. if you connect to google fit. You can write a dairy, share results with your doctor, you can add a family member's medicines or add someone to help you with medicines.	Android, iOS	-	No	Yes	Yes	4	4
**SE 19**	Livsmedelsdatabasen	https://play.google.com/store/apps/details?id=com.hackspett.ldb	General public	HEALTH	Not disease-specific	The app provides information about food and what it contains e.g. energy, fat, vitamins etc.	Android, iOS	Yes	-	Yes	Yes	3	3
**SE 20**	Pillerkollen	https://play.google.com/store/apps/details?id=com.pillerkollen.pillkoll	General public	HEALTH	Not disease-specific	It is an app where you can search (or scan) a medicine and then get information about it; Side effects, it is bad if a particular medicine is not recommended to take among older people.	Android, iOS	Yes	-	Yes	Yes	4	4
**SE 21**	STROKE-Riksförbundet	https://play.google.com/store/apps/details?id=com.appmachine.p3132FJ	General public	HEALTH	Stroke	Information about how to recognise a stroke. In addition, a description of a test that you can perform to see if a person has had a stroke.	Android, iOS	Yes	-	Yes	Yes	4	5
**SE 22**	Min doktor-läkarbesök online	https://play.google.com/store/apps/details?id=se.mindoktor.android	General public	HEALTH	Not disease-specific	A health care centre via app. You can get a doctor appointment; you describe your symptoms and then a doctor goes through your "case" and responds. Through text message, telephone call or video link the	Android, iOS	Yes	Yes	No	Yes	5	5

Country	Name	Link	Target	Category	Disease/ condition	Description	Op. system	Reliability	Privacy	Registration	Connection	MARS	Score
						doctors can set a diagnosis and send a prescription to the mobile phone.							
**SE 23**	Mina recept apoteket	https://play.google.com/store/apps/details?id=se.apoteket.recept	General public	HEALTH	Not disease-specific	App that helps you keep track on your prescribed medicines. You can get an overview over your prescribed medicines and high-cost status. You can see the stock and choose the cheapest alternative, see where my medicines are in stock. Advice via telephone while ordering medicine via the app. Chat with pharmacist.	Android, iOS	Yes	Yes	No	Yes	4	5
**SE 24**	Kry - träffa läkare online	https://play.google.com/store/apps/details?id=se.kry.android	General public	HEALTH	Not disease-specific	Doctor online service through video-communication. Advice, prescription and referral. Log in with bank ID.	Android, iOS	Yes	Yes	Yes	Yes	5	5
**SE 25**	Apotek hjärtat	https://play.google.com/store/apps/details?id=com.presencekit.apotek.se	General public	HEALTH	Not disease-specific	Prescribed medicines, order, advice, opening hours, find pharmacy.	Android, iOS	-	Yes	Yes	Yes	4	3
**SE 26**	Vårdappen Information &råd	https://play.google.com/store/apps/details?id=com.firconesoftware.vardappen	General public	HEALTH	Not disease-specific	The developer is a private company. It is an information app about illnesses and conditions, vaccinations, laws and rights in healthcare, self-care, etc.	Android, iOS	-	-	Yes	Yes	3	3
**SE 27**	Senioröver vakare	https://itunes.apple.com/se/app/seniorovervakare-aldrevard/id1095716826?mt=8	Carers & care recipients	TECHNOLOGIES	Not disease-specific	The app provides a platform for video-communication and interaction between the carer and the care recipient.	iOS	Yes	Yes	Yes	Yes	3	4

Country	Name	Link	Target	Category	Disease/ condition	Description	Op. system	Reliability	Privacy	Registration	Connection	MARS	Score
SE 28	Teckenspråk	https://play.google.com/store/apps/details?id=com.hiitti.tecken	General public	TECHNOLOGIES	Not disease-specific	The app teaches you the sign language in Swedish. You can get demonstration of the alphabet or write a text (or speak) and get it translated into sign language.	Android, iOS	No	-	Yes	Yes	4	3

WEBSITES

Country	Name	Link	Target	Category	Disease/condition	Description	Reliability	Privacy	Registration	SUS	Score
IT43	We are Parky	https://www.facebook.com/groups/wearepar ky/	Carers & care recipients	CONTACTS	Neurodegenerative	This Facebook group pursues the following aims: improving the recognition and integration of patients with Parkinson into society; promoting research of any form of care that might improve the quality of life of Parkinson's patients and those close to them, and letting people with Parkinson and their relatives to communicate and share experiences with people who are living the same experience.	-	Yes	Yes	-	4
IT44	Malati Alzheimer giovani	https://www.facebook.com/alzheimermalatigiovani/	Carers & care recipients	CONTACTS	Neurodegenerative	This FB page is dedicated to people with early onset Alzheimer's disease. It pursues different aims: to improve the recognition of early onset Alzheimer's disease and to provide information on how to recognize the warning symptoms, and the difference between the age-related changes and the signs of illness. It includes several testimonies.	Yes	-	No	-	4
IT45	Amministratori di Sostegno	http://amministratoridisostegno.com/	Carers & care recipients	CONTACTS	Not disease-specific	This website gives information on the support administrator's role (a sort of a guardian): who to contact, when and where submitting an application. It	-	No	No	78	3

Country	Name	Link	Target	Category	Disease/condition	Description	Reliability	Privacy	Registration	SUS	Score
						is possible to download a short handbook with all the information.					
**IT46**	Essere caregiver	https://www.facebook.com/caregiverfamiliare	Carers	CONTACTS	Not disease-specific	This Facebook group gives information on different initiatives addressed to caregivers and their families, and promotes contact between them.	Yes	Yes	No	-	4
**IT47**	Progetto Mondiale Asma	http://ginasma.it/	Carers & care recipients	CONTACTS	Respiratory	This website collects information on diagnosis and treatment of asthma.	-	No	No	75	3
**IT48**	A.IT.A - Associazioni Italiane Afasici	https://it-it.facebook.com/AITAFederazione/	Carers & care recipients	CONTACTS	Stroke, Neurodegenerative	This Facebook group has two aims: raising awareness on aphasia and promoting contact between people with aphasia and their families.	-	Yes	No	-	3
**IT49**	Disabili.com	https://www.disabili.com/	General public	CONTACTS	Not disease-specific	This website collects information on various aspects of disability: law, work, school and education, useful products and technologies, medicine and health, architectural barriers and mobility, culture, holidays, leisure, sports, family and friends. It gives people the chance to contact specialists to ask questions.	-	Yes	No	88	5
**IT50**	Diabete.net	http://www.diabete.net/	Carers & care recipients	HEALTH	Diabetes	This website collects general information on diabetes and anything concerning being a person with diabetes. It also gives practical hints to face daily challenges related to the disease.	-	No	No	93	4
**IT51**	diabete.com	http://www.diabete.com/	Care recipients	HEALTH	Diabetes	This website provides detailed information about diabetes, lifestyles, and practical guidance for managing the disease and its complications. It also includes interactive tools, allowing visitors to test their knowledge about the disease, and to interact with experts. The site is managed by a Scientific Committee composed by physicians, psychologists and nutritionists.	Yes	Yes	No	78	5

Country	Name	Link	Target	Category	Disease/condition	Description	Reliability	Privacy	Registration	SUS	Score
**IT52**	nientemale.it	http://www.nientemale.it	General public	HEALTH	Musculoskeletal	This website provides information about chronic and acute pain (mainly of musculoskeletal origin), and its management. It includes a section specifically devoted to pain in elderly people and a news section collecting information about the most recent findings on pain therapy.	Yes	Yes	No	78	4
**IT53**	myperfectdish.it	http://www.myperfectdish.it/it/index.aspx	Carers & care recipients	HEALTH	Neurodegenerative	This platform gives to patients and their carers useful information to create a varied, tasty and balanced diet for people living with Parkinson's disease. The platform is endorsed by the European Parkinson's Disease Association (EPDA).	Yes	Yes	Yes	83	4
**IT54**	Chat yourself	https://www.facebook.com/chatyourselfitalia/	Care recipients	HEALTH	Neurodegenerative	This Chabot supports patients at early stages of Alzheimer disease by memorizing useful information about their daily routines. It will remind the interlocutor about the things the person can forget such as: to take medicine, call relatives and friends, remember his/her address, sending the relevant information via chat.	Yes	Yes	Yes	-	4
**IT55**	anzianievita.it	http://www.anzianievita.it/	General public	HEALTH	Neurodegenerative, Incontinence	This website provides general information about health and wellbeing of the elderly, in particular those suffering from dementia. It includes many sections (e.g. nutrition, hygiene management, utilities) with the aim to provide caregivers with basic yet reliable information about topics related to the care.	Yes	-	No	78	3
**IT56**	farmacoecura.it	http://www.farmacoecura.it/	General public	HEALTH	Not disease-specific	This website provides basic information about many medical conditions/diseases (including the main neurodegenerative diseases of elderly) with the aim to provide the public with reliable and quality information on health-related topics. The site is certified by Health on the Net Foundation (HoN).	Yes	-	No	78	5

Country	Name	Link	Target	Category	Disease/condition	Description	Reliability	Privacy	Registration	SUS	Score
**IT57**	aimac.it	https://www.aimac.it/	Carers & care recipients	HEALTH	Cancer	This website provides information for cancer patients and their carers. It includes information sheets about the different cancer types and their treatments. It also includes a complete list of national cancer hospitals and a forum group for patients and families. The site is certified by Health on the Net Foundation (HoN).	Yes	Yes	Yes	80	5
**IT58**	Fondazione ANT	http://www.ant.it/	General public	HEALTH	Cancer	This website collects information on cancer services. It is possible to download a handbook, targeted at caregivers, which contains useful advice to take care of cancer patients at home.	-	No	No	73	3
**IT59**	AIRC	http://www.airc.it/	General public	HEALTH	Cardiovascular, Cancer, Incontinence	This website gives information on facing cancer: screening, treatments, early diagnosis, examinations. It also gives information on scientific researches about cancer.	-	No	No	93	5
**IT60**	Informcare	http://eurocarers.org/informcare/?lang=it	Carers	HEALTH	Cardiovascular, Stroke, Neurodegenerative, Musculoskeletal, Diabetes, Sense, Incontinence	First European web-platform for informal carers, developed in the framework of the EU-funded Innovage project. InformCare provides informal carers from 27 EU member states with key information on their rights and entitlements as well as advice on how to best deliver and cope with their caregiving activities. Care professionals and employers from a selection of countries, are also able to access information on how to best support carers and meet their needs.	Yes	Yes	No	88	5
**IT61**	parkinson.it	http://www.parkinson.it/aip	Care recipients	HEALTH	Neurodegenerative	This website provides detailed information about Parkinson disease, its symptoms and complications, including tips for those who assist the sick person. It also provides updates on the most recent findings about the disease.	Yes	Yes	Yes	70	4
**IT62**	aims.it	http://www.aism.it/	Care recipients	HEALTH	Neurodegenerative	This website is promoted by the Italian Multiple Sclerosis Society (AISM) and provides a complete	Yes	-	No	80	5

Country	Name	Link	Target	Category	Disease/condition	Description	Reliability	Privacy	Registration	SUS	Score
						overview of the disease and its management, including suggestions for caregivers, and information about legal/advocacy issues. The site is certified by Health on the Net Foundation (HoN).					
**IT63**	osservatoriomalattierare.it	http://www.osservatoriomalattierare.it/	Care recipients	HEALTH	Neurodegenerative, Cancer	This website mainly provides information about rare diseases (including rare cancers), but it also includes specific sections devoted to chronic diseases (such as Parkinson and Alzheimer diseases) and information on disability support, exemptions and rights, and home care therapies. The site is certified by Health on the Net Foundation (HoN).	Yes	Yes	No	80	4
**IT64**	Caregiver Familiare	http://www.caregiverfamiliare.it/	Carers	CONTACTS	Not disease-specific	This website gives information on caregiver, presents projects, and provides different contents, such as articles and documents. It provides a virtual space in which caregivers can tell their experiences to share with others who are living similar situations.	Yes	Yes	No	85	4
**IT65**	aliceitalia.org	http://www.aliceitalia.org/	Carers & care recipients	HEALTH	Stroke	This website provides detailed information about stroke, its consequences and the rehabilitation process. It also includes a specific section devoted to patients 'right (e.g. disability support, exemptions, allowances), and a national list of rehabilitation centres and stroke units.	Yes	Yes	No	83	5
**IT66**	fondazioneveronesi.it/magazine	http://www.fondazioneveronesi.it/magazine	General public	HEALTH	Stroke, Neurodegenerative, Cancer	This online magazine of the Umberto Veronesi Foundation aims to provide people with reliable information on health-related issues. It includes specific sections devoted to oncology, cardiology, neurology and nutrition, and a number of informative tools for users (e.g. glossary, self-medication, prevention checks, etc.).	Yes	Yes	Yes	80	4

Country	Name	Link	Target	Category	Disease/condition	Description	Reliability	Privacy	Registration	SUS	Score
CY11	Anticancer Society	http://www.anticancersociety.org.cy/anticancer1/page.php?pageID=1	Care recipients	CONTACTS	Cancer	This website provides information on the activities and services of the non-profit association in Cyprus	Yes	Yes	No	78	4
CY12	Cyprus antirheumatism Association	http://www.rheumatism.org.cy	Carers & care recipients	CONTACTS	Musculoskeletal	This website provides information on the activities and services of the non-profit association. Moreover, there are links on brochures of the association providing info on rheumatoid diseases	Yes	-	No	78	4
CY13	Ithaki Association	http://ithakicyprus.com	Carers	CONTACTS	Neurodegenerative	Ithaki's association website provides information on events, services and dementia. Ithaki, non-profit association, is based in Limassol, Cyprus	Yes	-	No	72	2
CY14	Cyprus Alzheimer's Association	http://cyprusalzheimerassociation.weebly.com	Carers	CONTACTS	Not disease-specific	Cyprus Alzheimer Association website provides information on events, services and dementia. The Association covers all 5 regions of Cyprus.	Yes	-	No	72	2
CY15	Informcare	http://eurocarers.org/informcare/?lang=cy	Carers	HEALTH	Not disease-specific	Innovage platform provided information for carers. The platform has been developed in 27 EU Member states and provides national specific information and general information on older people chronic diseases and the management of caregiving. The mobile interface appears in English and the language option is not visible directly.	Yes	Yes	No	70	4
CY16	ELEANA	http://www.arthritis.org.gr	Care recipients	CONTACTS	Musculoskeletal	This website provides information on the activities and services of the no-profit association. Moreover, there are links on brochure and videos providing info on rheumatoid diseases.	Yes	-	No	78	4
CY17	Alzheimer's	www.alzheimerathens.gr	Carers	CONTACTS	Neurodegenerative	Athens Alzheimer Association website provides information on events, services and dementia in	Yes	-	No	82	4

Country	Name	Link	Target	Category	Disease/condition	Description	Reliability	Privacy	Registration	SUS	Score
	Athens Association					Greece. Carers could find valuable material on the disease.					
**CY18**	Greek Multiple Sclerosis Society	http://www.gmss.gr	Carers & care recipients	CONTACTS	Neurodegenerative	Website of the Greek Multiple Sclerosis Society, providing information on services, events, etc.	Yes	Yes	No	78	3
**CY19**	Greek Association of Palliative Care	http://grpalliative.gr/el/	Care recipients	CONTACTS	Not disease-specific	The website represents a non-for-profit association working with patients and their families provides information on the treatment of chronic pain and the end of life care.	Yes	-	No	70	4
**CY20**	heart failure matters	http://www.heartfailurematters.org/el_GR	General public	HEALTH	Cardiovascular	A portal for heart failure, providing all the necessary information for people with heart diseases: understanding of heart disease, treatment options, health care professionals' options, living with heart disease, FAQ, and questions for the doctor.	Yes	Yes	No	70	4
**CY21**	Trainyourbrain	www.trainyourbrain.gr	Carers & care recipients	HEALTH	Neurodegenerative	Trainyourbrain has been developed by health professionals to support cognitive training of people with dementia and their families.	Yes	-	No	82	4
**CY22**	Nestor	http://www.nstr.gr	Carers & care recipients	CONTACTS	Neurodegenerative	Nestor association website provides information on events, services and dementia in Greece. Carers could find valuable material on the disease.	Yes	-	No	68	4
**GR12**	Doctoranytime	https://www.doctoranytime.gr	Carers & care recipients	CONTACTS	Not disease-specific	This website manages the doctor appointments in private and social secure services. It is valid and the comments provide a sense of validation for the users.	-	-	Yes	72	4
**GR13**	50plus Hellas	http://www.50plus.gr	General public	CONTACTS	Not disease-specific	A website providing information for older people and the available services aiming to active and healthy ageing. The non-profit association works on the domain on healthy and active ageing, organizing trainings to support lifelong learning of older people.	Yes	-	No	68	3

Country	Name	Link	Target	Category	Disease/condition	Description	Reliability	Privacy	Registration	SUS	Score
**GR14**	life line Hellas	http://www.lifelinehellas.gr	General public	CONTACTS	Not disease-specific	Life line Hellas is an innovative association working in the field of older people and telemedicine, including red button service, silver alert, for older people missing, national line for older people, and other services supporting older people in need.	Yes	-	No	72	5
**GR15**	Informcare	http://eurocarers.org/informcare/?lang=el	Carers	HEALTH	Not disease-specific	Innovage platform provided information for carers. The platform has been developed in 27 EU Member States and provides national specific information and general information on the older people chronic diseases and the management of caregiving. The mobile interface appears in English and the language option is not visible directly.	Yes	Yes	No	70	4
**GR16**	ELEANA	http://www.arthritis.org.gr	Care recipients	CONTACTS	Musculoskeletal	This website provides information on the activities and services of the no-profit association. Moreover, there are links on brochure and videos providing info on rheumatoid diseases.	Yes	-	No	78	4
**GR17**	Alzheimer's Athens Association	www.alzheimerathens.gr	Carers	CONTACTS	Neurodegenerative	Athens Alzheimer Association website provides information on events, services and dementia in Greece. Carers could find valuable material on the disease.	Yes	-	No	82	4
**GR18**	Greek Multiple Sclerosis Society	http://www.gmss.gr	Carers & care recipients	CONTACTS	Neurodegenerative	Website of the Greek Multiple Sclerosis Society, providing information on services, events, etc.	Yes	Yes	No	78	3
**GR19**	Greek Association of Palliative Care	http://grpalliative.gr/el/	Care recipients	CONTACTS	Not disease-specific	The website represents a non-for-profit association working with patients and their families provides information on the treatment of chronic pain and the end of life care.	Yes	-	No	70	4

Country	Name	Link	Target	Category	Disease/condition	Description	Reliability	Privacy	Registration	SUS	Score
**GR20**	heart failure matters	http://www.heartfailurematters.org/el_GR	General public	HEALTH	Cardiovascular	A portal for heart failure, providing all the necessary information for people with heart diseases: understanding of heart disease, treatment options, health care professionals' options, living with heart disease, FAQ, and questions for the doctor.	Yes	Yes	No	70	4
**GR21**	Trainyourbrain	www.trainyourbrain.gr	Carers & care recipients	HEALTH	Neurodegenerative	Trainyourbrain has been developed by health professionals to support cognitive training of people with dementia and their families.	Yes	-	No	82	4
**GR22**	Nestor	http://www.nstr.gr	Carers & care recipients	CONTACTS	Neurodegenerative	Nestor association website provides information on events, services and dementia in Greece. Carers could find valuable material on the disease.	Yes	-	No	68	4
**PT34**	Alzheimer Portugal	http://alzheimerportugal.org/pt/	General public	CONTACTS	Neurodegenerative	The websites aims to call attention to Alzheimer Diseases by providing info about on-going national and local information campaigns on Alzheimer; informing about the importance of early diagnosis, the importance of the role of general practitioners in the detection of signs of dementia, the role of caregivers and recognizing their specific needs and rights; on-going training actions for caregivers.	Yes	-	No	82	4
**PT35**	SOS Demências	http://www.sosdemencias.com	General public	CONTACTS	Neurodegenerative	SOS DEMENTIA is a platform directed to care recipients, carers that deal with patients that have neurodegenerative diseases and people interested in this theme. It provides information about the diseases provides news and informs about other websites.	Yes	-	No	78	4
**PT36**	Informcare	http://eurocarers.org/informcare/?lang=pt-pt	Carers	CONTACTS	Not disease-specific	First European web-platform for informal carers, developed in the framework of the EU-funded Innovage project. InformCare provides informal carers from 27 EU member states with key information on their rights and entitlements as well as advice on how to best deliver and cope with their caregiving activities. Care professionals and employers from a selection of	Yes	Yes	No	90	5

Country	Name	Link	Target	Category	Disease/condition	Description	Reliability	Privacy	Registration	SUS	Score
						countries, are also be able to access information on how to best support carers and meet their needs.					
**PT37**	mylinked care	http://www.mylinkedcare.com	General public	HEALTH	Not disease-specific	The mylinkedcare app is a platform that brings together all information concerning health. It can be accessed using a computer and it works on every operating systems and browsers. It enables users to record biometric data, such as blood pressure or temperature, and synchronize their physical activity. If the users' doctors use the platform as well, they can have access to all the introduced data. This app has also a feature that enables searching for doctors according to their specialties, seeing prices charged and availability and making an appointment. Furthermore, users can also buy medication and choose home-delivery.	Yes	Yes	Yes	80	4
**PT38**	Cuidadores + perto	https://www.facebook.com/cuidadoresmaisperto/?fref=ts	Carers	WELL-BEING	Neurodegenerative	The project aims to contribute to the quality of life and decrease the social isolation of caregivers of people with dementia, by promoting a training and support network of caregivers, based on the proximity relations and the sponsorship of more experienced caregivers.	Yes	-	No	90	4
**SE29**	Anhörigstödet	www.anhorigstodet.se	Carers	HEALTH	Not disease-specific	The website aims at supporting carers of people with addiction to alcohol, drugs or gambling. It provides information and a forum for exchanging carers' stories and enabling peer support.	Yes	Yes	Yes	85	4
**SE30**	Fass	www.fass.se	General public	HEALTH	Not disease-specific	The website aims to provide information about the availability and characteristics of pharmaceutical products. The user can search for detailed information on a certain medicine, check the glossary and find the availability across pharmacies in the country.	Yes	Yes	No	75	3
**SE31**	Cancerkompisar	http://www.cancerkompisar.s	Carers	CONTACTS	Cancer	The website aims to bring carers of patients with carers together, they help match carers in similar situations.	Yes	-	No	93	5

Country	Name	Link	Target	Category	Disease/condition	Description	Reliability	Privacy	Registration	SUS	Score
		e/bloggar/ambassadorsbloggen				They have blogs and an online forum for carers of patients with cancer.					
**SE32**	Alzheimer.bloggo.nu	http://alzheimer.bloggo.nu/	Carers & care recipients	CONTACTS	Neurodegenerative	This is a private blog. The blogger is an older woman with Alzheimer's disease who writes about her everyday life.	-	-	No	93	4
**SE33**	Anhörigas riksförbund	http://anhorigasriksforbund.se/	Carers	CONTACTS	No	The developer is a national carer organisation. Website is targeted to carers. Provides information, news etc.	Yes	-	No	98	5
**SE34**	Nka	http://www.anhoriga.se/fragor-och-svar/fraga-200/	Carers & care recipients	CONTACTS	Not disease-specific	The developer is a national competence centre concerning carers, care and caring. Website provides information about carers and caring, online lectures and online education (professionals mostly).	Yes	-	No	93	4
**SE35**	Funktiosnhinder.se	www.funktionshinder.se	Carers & care recipients	CONTACTS	Not disease-specific	The website is a forum for persons with disabilities.	-	-	Yes	70	4
**SE36**	seniorval.se	www.seniorval.se	Carers & care recipients	CONTACTS	Not disease-specific	The website is a search and information service regarding living arrangements, service and care regarding seniors and carers. The purpose is to help seniors and their carers in decisions concerning care and living arrangements. They also provide tips and guides for e.g. meeting with the need's assessor, which living arrangement to choose etc.	-	-	No	75	4
**SE37**	äldrekontakt	www.aldrekontakt.se	Care recipients	CONTACTS	Not disease-specific	The developer is a non-profit organisation and sponsors. They organise different events for older people who are alone and seldom meet friends and family to get in contact with others.	Yes	-	No	80	3
**SE38**	Senioren	http://www.senioren.se/omvarld-	General public	CONTACTS	Not disease-specific	The developer is an organisation for seniors in Sweden. The website provides news concerning senior's daily	Yes	-	No	85	3

Country	Name	Link	Target	Category	Disease/condition	Description	Reliability	Privacy	Registration	SUS	Score
		ekonomi/vard-omsorg/sa-hjalper-du-en-anhorig-med-demens/				life and ageing. The purpose is to create debate and opinion.					
**SE39**	Apoteksinfo	https://apoteksinfo.nu	General public	CONTACTS	Not disease-specific	On the website, you can do a search concerning opening hours for all pharmacies in Sweden.	-	-	No	100	5
**SE40**	sos alarm	https://www.sosalarm.se/	General public	CONTACTS	Not disease-specific	The developer is SOS alarm who develops existing and new services from a general crisis management perspective, for example which numbers to call in different crises, 112 for example.	Yes	-	No	80	3
**SE41**	pensionsmyndigheten	www.pensionsmyndigheten.se	General public	CONTACTS	Not disease-specific	The website is providing information about your retirement and financial planning concerning retirement. You can register to have "your own page" if you want to. You can get tips about how to manage your retirement allowance.	Yes	-	No	70	3
**SE42**	äldreomsorgsbloggen	http://www.aldreomsorgsbloggen.se/	General public	CONTACTS	Not disease-specific	The website is a blog regarding topics about ageing and older people. Provides news in the area of older people and has a section targeted at carers.	-	-	No	90	3
**SE43**	Teletal	http://www.teletal.se/	Care recipients	TECHNOLOGIES	Not disease-specific	The website informs about a service where you can get support and interpretation support during a telephone call. Teletal is a free and national service where you get support from a third person in the conversation. You don´t have to book the service just call.	-	-	No	95	3
**SE44**	Hjälpmedelsteknik Sverige	www.hjalpmedel.se	General public	TECHNOLOGIES	Not disease-specific	The developer is a private company selling different kinds of assistive devices. The website provides a description of different assistive devices and has a web shop.	Yes	-	No	78	3
**SE45**	Stroke.se	www.stroke.se	General public	HEALTH	Cardiovascular, Stroke	The developer seems to be a private company. No description about the company Health Care Media	-	-	No	83	3

Country	Name	Link	Target	Category	Disease/condition	Description	Reliability	Privacy	Registration	SUS	Score
						behind the website. Provides information about stroke. One section about aphasia.					
**SE46**	Hjärt-och lungfonden	www.hjart-lungfonden.se	General public	HEALTH	Cardiovascular, Stroke, Respiratory, Diabetes	The developer is an association. They collect donations to support research about heart and lung diseases. Website provides information about heart and lung diseases. They have a section targeting carers.	Yes	-	No	80	4
**SE47**	Svensk dystoniförening	www.dystoni.se	Care recipients	HEALTH	Musculoskeletal	Website provides information on a rather rare illness called dystonia; it is a neurological disease affecting muscles and movement. Have a section targeting carers.	Yes	-	No	95	4
**SE48**	Svenskt Demenscentrum	www.demenscentrum.se	General public	HEALTH	Neurodegenerative	The developer is a national centre for knowledge development within the area of dementia. They provide information about dementia and they have a section targeting carers and living with dementia.	Yes	-	No	95	4
**SE49**	Infoteket om funktionshinder	http://www.lul.se/sv/Kampanjwebbar/Infoteket/	Carers & care recipients	HEALTH	Neurodegenerative, Mental	The website provides information about disabilities.	-	-	No	85	3
**SE50**	Pfizer hälsa	https://www.pfizerhalsa.se	Carers & care recipients	HEALTH	Not disease-specific	The website is targeted at carers and patients. Gives information about treatment and health information.	-	-	No	75	3
**SE51**	Läkemedelsverket /Medical Products Agency	https://lakemedelsverket.se/malgrupp/Allmanhet/Sjukdom-och-behandling/Behandlingsrekommendationer---listan/Behandli	General public	HEALTH	Not disease-specific	The developer is the Medical Products Agency. The purpose with the website is to contribute to the safe and effective use of medicines, drugs, cosmetics and medical devices in society.	Yes	Yes	No	73	3

Country	Name	Link	Target	Category	Disease/condition	Description	Reliability	Privacy	Registration	SUS	Score
		ng-och-bemotande-vid-beteendemassiga-och-psykiska-symto									
**SE52**	Arlas kundportal	https://kund.arla.se	General public	HEALTH	Not disease-specific	The website provides food recipes. Contains a section with recipes adapted for seniors.	Yes	-	No	78	4
**SE53**	Habilitering och hälsa	www.habilitering.se	Carers & care recipients	HEALTH	Mental	The website provides information about disabilities in all ages, news, tips. They have a chat or telephone line where you can get advice. County council in Stockholm is the developer	Yes	-	No	73	4
**SE54**	Autism- och aspergerförbundet	http://www.autism.se/	Carers & care recipients	HEALTH	Mental	The developer is an association. Website provides, information, news.	Yes	-	No	88	3
**SE55**	Parkinsonförbundet	www.parkinsonforbundet.se	Carers & care recipients	HEALTH	Neurodegenerative	The developer is an association. Website provides information about Parkinson's disease. They have a helpline for patients and carers. They have a section targeted at carers.	Yes	-	No	90	3
**SE56**	Alzheimer Sverige	www.alzheimersverige.se	Carers & care recipients	HEALTH	Neurodegenerative	Website provides information about Alzheimer's disease. They have a section especially targeted to carers. They have a helpline.	Yes	-	No	98	3
**SE57**	Neuroförbundet	www.neuroforbundet.se	Carers & care recipients	HEALTH	Neurodegenerative	The developer is an organisation. Website provides information about different neurological diseases. They have a blog where members can comment and they also have a Forum but registration is needed.	Yes	-	No	90	3
**SE58**	Handikapptips	https://handikapptips.wordpress.com/	Carers & care recipients	HEALTH	Neurodegenerative	This is a private blog about dementia and people with disabilities and for people with dementia and disabilities. Provides tips and ideas.	-	-	No	88	4

Country	Name	Link	Target	Category	Disease/condition	Description	Reliability	Privacy	Registration	SUS	Score
**SE59**	Demensförbundet	www.demensforbundet.se	Carers & care recipients	HEALTH	Neurodegenerative, Mental	The developer is an association. Website provides information about different types of dementia and also information needed in relation to dementia e.g. different aids, news etc.	Yes	-	No	98	5
**SE60**	Funkaportalen	www.funkaportalen.se	Carers & care recipients	HEALTH	Not disease-specific	The website provides information about disabilities, news, tips, thoughts and ideas. The developer is a foundation.	-	-	No	68	4
**SE61**	Hörselskada.se	http://hörselskada.se/hjalpmedel/	Care recipients	HEALTH	Not disease-specific	The website developer is a company. The website provides information and news about hearing impairment and hearing impairment aids. They also have a social forum where you can read posts but if you want to write you have to register.	-	-	No	78	3
**SE62**	Hörsellinjen	https://horsellinjen.se/fakta-och-rad/horhjalpmedel/tips-om-smarta-horhjalpmedel/	Care recipients	HEALTH	Not disease-specific	The website provides information about hearing impairment. They also have a counselling service, via phone or electronically.	-	-	No	93	3
**SE63**	Privatmedicin Information om sjukdomar för privatpersoner	www.privatmedicin.se	General public	HEALTH	Not disease-specific	It is not very clear who the developer "privatmedicin" is. The website provides information about illnesses targeting the public and a "medicinbook" with the most common illnesses, symptoms and treatments. They also have a forum where you can ask questions if you are registered.	-	-	No	75	3
**SE64**	Netdoktor	http://www.netdoktor.se/	General public	HEALTH	Not disease-specific	The developer is Netdoktor. Financed by sponsors and advertisements. Doctors have written material and are answering questions from members. If you log in you can ask questions to a doctor and use a forum. You can	Yes	Yes	No	78	4

Country	Name	Link	Target	Category	Disease/condition	Description	Reliability	Privacy	Registration	SUS	Score
						also a couple of times per month use a chat and ask questions to a doctor.					
**SE65**	1177 Vårdguiden	https://www.1177.se	General public	HEALTH	Not disease-specific	The developer is 1177 Vårdguiden, the Swedish county councils and regions. Website provides information about different illnesses and conditions. They have a section targeting carers.	Yes	-	No	98	5
**SE66**	Koll på läkemedel	http://www.kollpalakemedel.se/guider-rad/for-forskrivare/hypertoni/	General public	HEALTH	Not disease-specific	The developers are two senior organizations and Apoteket, which are driven by the government, and they sell medications, give advice concerning medications etc. The website provides information about medications e.g. which medications are not compatible. The purpose with the website is to decrease the over prescription and wrong prescriptions of medicines/drugs to older people.	Yes	Yes	No	78	4
**SE67**	Afasiförbundet i Sverige	www.afasi.se	Carers & care recipients	HEALTH	Stroke	The developer is an association. Website provides information about aphasia, news etc	Yes	-	No	95	4
**SE68**	Hjärnfonden	www.hjarnfonden.se	General public	HEALTH	Stroke, Neurodegenerative, Cancer, Musculoskeletal, Mental	Website provides information about different brain diseases/conditions. The provider is a foundation.	Yes	-	No	93	3
**SE69**	Rehabvalet	http://www.rehabvalet.se	Carers & care recipients	HEALTH	Stroke, Neurodegenerative, Musculoskeletal, Mental	It is not clearly stated who is behind the website. There is no information "about us". However, the website provides information about rehabilitation, about different support services, about some specific diseases or conditions.	-	-	No	73	4
